# Synthesis, Structure and Reactivity of a Cyapho(dicyano)methanide Salt

**DOI:** 10.1002/anie.202208921

**Published:** 2022-08-18

**Authors:** Chenyang Hu, Jose M. Goicoechea

**Affiliations:** ^1^ Department of Chemistry University of Oxford Chemistry Research Laboratory 12 Mansfield Road Oxford OX1 3TA UK

**Keywords:** Cyanide, Cyanoform, Cyaphide, Phosphorus, Tricyanomethanide

## Abstract

We describe the synthesis of a cyapho(dicyano)methanide salt, [K(18‐crown‐6)][C(CN)_2_(CP)], from reaction of [Na(18‐crown‐6)][PH_2_] (18‐crown‐6=1,4,7,10,13,16‐hexaoxacyclooctadecane) with 1,1‐diethoxy‐2,2‐dicyanoethylene (EtO)_2_C=C(CN)_2_. The reaction proceeds through a Michael addition‐elimination pathway to afford [Na(18‐crown‐6)][HP{C(OEt)=C(CN)_2_}]. Addition of a strong, non‐nucleophilic base (KHMDS) to this intermediate results in the formation of [K(18‐crown‐6)][C(CN)_2_(CP)]. Subsequent reactivity studies reveal that the cyapho(dicyano)methanide ion is susceptible to protonation with strong acids to afford the parent acid HC(CN)_2_(CP). The reactivity of the cyaphide moiety in [C(CN)_2_(CP)]^−^ was explored through coordination to metal centers and in cycloaddition reactions with azides.

## Graphical abstract

We describe the synthesis of the cyapho(dicyano)methanide ion, [C(CN)_2_(CP)]^−^, by reaction of sodium phosphanide with (EtO)_2_C=C(CN)_2_. This species can be readily protonated at the methanide carbon atom to afford the parent acid, HC(CN)_2_(CP), which readily decomposes at room temperature. The reactivity of the cyaphide moiety was also probed by reaction with nucleophiles and in cycloaddition reactions.

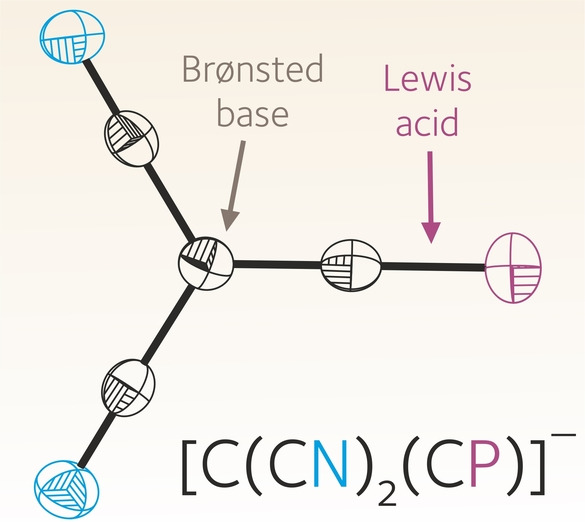

Tricyanomethane (or cyanoform; HC(CN)_3_) is a highly unstable acid (p*K*_a_=−5.1 in water) that was first observed by microwave spectroscopy in the gas phase,^[1]^ and only recently isolated in solution by Kornath and co‐workers.^[2]^ By contrast, its conjugate base, tricyanomethanide (TCM), is a well‐established weak organic base first synthesized from the reaction of potassium cyanate and malononitrile by Schmidtmann in 1896.^[3]^ Due to its extended conjugated π‐system, TCM is a resonance stabilized pseudo‐halide with a planar geometry.^[4]^ The lone pairs on the three nitrogen atoms allows TCM to coordinate to metal centers, a property which has seen it used as a building block in coordination polymers.^[5]^ Coordination to main group Lewis acids can also be used to access weakly coordinating anions.^[6]^ Moreover, the high stability and delocalized charge of this ion allows access to ionic liquids in which TCM is combined with a bulky counter‐cation. Such ionic liquids have been used in a number of applications including CO_2_ separation, the synthesis of dye‐sensitized solar cells and the extraction of aromatic hydrocarbons.^[7]^

Nitrile‐containing inorganic anions of generic formula [E(CN)_*n*_]^−^ (E=p‐block element; Figure 1) are a relatively large family, many of which are stable on account of the electron‐withdrawing properties of the cyano‐group and its ability to stabilize a multitude of resonance structures. For example, the TCM ion can be thought of as being composed of a central group 14 element (a carbon atom) surrounded by three cyano‐groups. An analogous compound with a central group 15 element would be dicyanamide, [N(CN)_2_]^−^, which was first synthesized by Madelung and Kern in 1922.^[8]^ Like TCM, dicyanamide has also found uses in coordination polymers and ionic liquids.[^5d^, ^9^] A group 16 version of this class of ions is cyanate OCN^−^, which is an archetypal inorganic anion with a myriad of applications, synthesized industrially by heating of a mixture of sodium carbonate and urea.^[10]^ Due to the importance of these nitrile‐containing small anions, the synthesis of heavier analogues in which one or more of the nitrogen atoms is replaced by a heavier pnictogen element has attracted significant interest. For example, Becker first synthesized the lithium salt of the 2‐phosphaethynolate anion, OCP^−^, in 1992,^[11]^ and in recent years this ion has been shown to be a valuable building block for the synthesis of an incredibly broad range of phosphorus‐containing compounds.^[12]^ Heavier analogues of this ion, such as ECP^−^ (E=S, Se),^[13]^ and OCAs^−^, have also been synthesized.^[14]^ Analogues of the dicyanamide ion where one of the nitrogen atoms has been replaced by heavier elements have also been reported. The dicyanophosphide ion, [P(CN)_2_]^−^, was first synthesized by Schmidpeter in 1977.^[15]^ However, due to difficulties associated with its synthesis, its reactivity was not explored until forty years later when Macdonald and Liu separately discovered new synthetic routes towards this ion.^[16]^ More recently, (cyapho)cyanamide, [N(CN)(CP)]^−^, was also synthesized by our group.^[17]^ The fact that heavier phosphorus‐containing analogues of the cyanate and dicyanamide ion are now synthetically available prompted us to explore the synthesis of a phosphorus‐containing tricyanomethanide ion. Herein, we describe the synthesis of the cyapho(dicyano)methanide [C(CN)_2_(CP)]^−^ anion employing a similar synthetic protocol as used for the synthesis of (cyapho)cyanamide, [N(CN)(CP)]^−^, and the 2‐phosphaehtynolate ion, PCO^−^.[^17^, ^18^]

**Figure 1:**
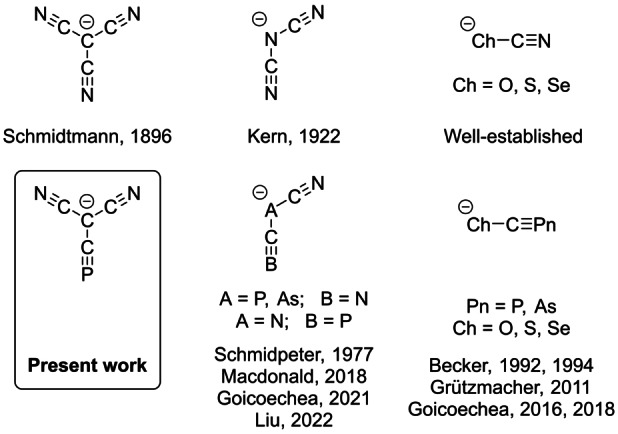
Selected nitrile‐containing inorganic anions and their heavier analogues.

In order to access our target ion, cyapho(dicyano)methanide, we identified 1,1‐diethoxy‐2,2‐dicyanoethylene (EtO)_2_C=C(CN)_2_ as a viable precursor since it contains the desired C(CN)_2_ moiety. The nitrile substituents on the ethylene also serve to polarize the C=C bond making it susceptible to nucleophilic attack by sodium phosphanide, NaPH_2_, as would be expected in a Michael addition. Elimination of two equivalents of ethanol following the Michael addition would generate our desired ion. Reaction of a THF solution of [Na(18‐crown‐6)][PH_2_] and (EtO)_2_C=C(CN)_2_ at −78 °C (Scheme 1) gave rise to a yellow solution after allowing the reaction mixture to slowly warm to room temperature overnight. *In situ* ^31^P NMR spectroscopy revealed a doublet at −66.3 ppm with a ^1^ *J*_H−P_ coupling constant of 166.5 Hz corresponding to [Na(18‐crown‐6)][HP{C(OEt)C(CN)_2_}]^−^ ([Na(18‐crown‐6)]**1**). **1** is a relatively weak anionic phosphide and can co‐exist with the ethanol that is generated in the reaction mixture. In contrast to other related species, such as the precursor to the (cyapho)cyanamide ion,^[17]^ **1** does not undergo further elimination of ethanol on standing. It decomposes before eliminating ethanol when heated to 50 °C for 6 hours. However, treatment of [Na(18‐crown‐6)]**1** with KHMDS (potassium hexamethyl disilazide) at 0 °C for 2 days afforded the target compound [K(18‐crown‐6)][C(CN)_2_(CP)] ([K(18‐crown‐6)]**2**, Scheme 1), which features a singlet in its ^31^P NMR spectrum at 27.1 ppm. [K(18‐crown‐6)]**2** is soluble in polar solvents (e.g. THF) and moderately soluble in apolar aromatics such as toluene and benzene.

**Scheme 1:**
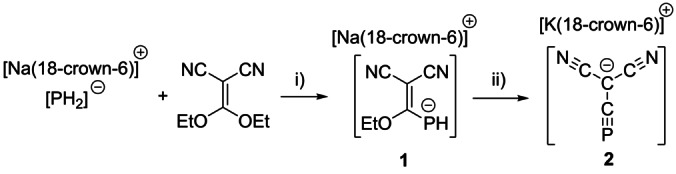
Synthesis of compound [Na(18‐crown‐6)]**1** and [K(18‐crown‐6)]**2**. i) THF, −78 °C to R.T. (yield 63 %); ii) THF, KHMDS, −78 °C to 0 °C (yield 64 %).

The structures of [Na(18‐crown‐6)]**1**⋅THF and [K(18‐crown‐6)]**2**⋅THF were confirmed by single crystal X‐ray diffraction (Figure 2).^[19]^ Both of the ions exhibit a planar geometry about the central carbon atom (Σ°: 360.0 for both **1** and **2**). The most notable structural change on going from **1** to **2** is the significant shortening of the C−P bond length which contracts from 1.742(2) to 1.553(2) Å, in line with the formation of a C≡P triple bond.^[20]^ The C−C bond lengths in **2** (1.390(3), 1.418(3) and 1.419(3) Å) all lie within the range between C−C single and C=C double bonds.^[21]^ Taken together, these bond metric data are indictive of high degree of conjugation. It is worth noting that the counter ion for **2** has changed from [Na(18‐crown‐6)]^+^ to [K(18‐crown‐6)]^+^. This is because KHMDS is used to facilitate the loss of an additional molecule of ethanol from **1**, and 18‐crown‐6 preferentially binds K^+^ over other alkali metal cations.^[22]^

**Figure 2:**
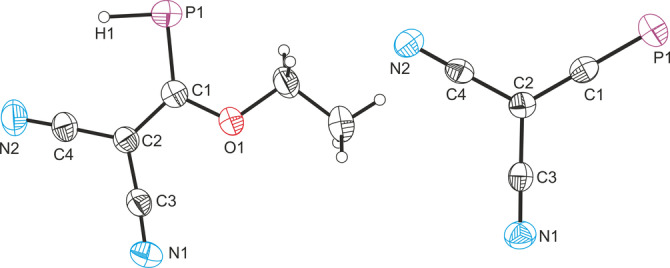
Single crystal X‐ray structures of the anionic components in [Na(18‐crown‐6)]**1**⋅THF (left) and [K(18‐crown‐6)]**2**⋅THF (right). Anisotropic displacement ellipsoids pictured at 50 % probability. Selected bond lengths [Å] and angles [°] **1**: P1−C1, 1.742(2); C1−C2 1.432(2); C1−O1, 1.355(2); C2−C3, 1.410(2); C2−C4, 1.404(2); C3−N1, 1.154(2); C4−N2, 1.150(2); P1−C1−O1, 121.67(12); P1−C1−C2, 127.78(13); C2−C1−O1, 110.55(14). **2**: P1−C1, 1.553(2); C1−C2 1.390(3); C2−C3, 1.418(3), C2−C4, 1.419(3), C3−N1, 1.149(3), C4−N2, 1.151(3); C1−C2−C3, 120.79(18), C1−C2−C4, 121.19(18); C3−C2−C4, 117.98(18).

In order to further understand the electronic structure of **2**, density functional theory (DFT) calculations (M06‐2X/def2‐SVP) were performed. The optimized geometry is in good agreement with the bond metrics obtained by X‐ray crystallography (e.g. P1−C2: 1.567 Å; C1−C2: 1.396 Å). Natural resonance theory (NRT) as implemented in NBO 7.0 confirmed extensive π‐orbital delocalization in **2**. All four major contributing resonance structures have a similar weighting (Figure 3). The heteroallene‐like isomer with negative charge at the terminal phosphorus atom has the greatest contribution (15.88 %) to the overall electronic structure, in contrast to the computed results for the tricyanomethanide ion (see Supporting Information). This is in line with the experimental observation that the C1−C2 bond length (1.390(3) Å) is slightly shorter than C2−C3 and C2−C4 (1.418(3) and 1.419(3) Å, respectively). This implies greater C−C double bond character between C1 and C2 than between C2−C3 and C2−C4. Second‐order perturbation theory suggests that back‐donation from the lone pair of C2 into the CP π* orbital is stronger than it is into the into CN π* orbitals (Figure S33). The nitrile group has long been known as a competent stabilizing functionality for a negative charge in α position, but in this case, theory suggests that cyaphide group is moderately better at delocalizing negative charge in α position, due to its lower lying C−P π* orbital.

**Figure 3:**
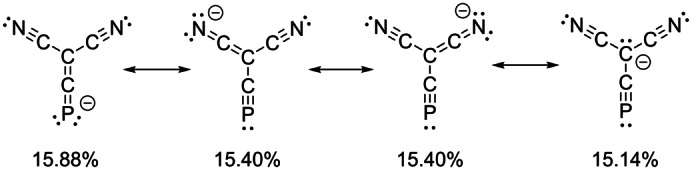
The four major resonance structures of **2** as predicted by natural resonance theory (NRT).

The reactivity of **2** was further explored with the aim of experimentally probing these bonding considerations. Due to the high degree of negative charge delocalization, **2** is a poor nucleophile compared to other phosphorus containing anions such as PCO^−^ or [N(CN)(CP)]^−^.[^12^, ^17^] It is inert towards moderate strength electrophiles such as Ph_3_GeCl and 1,3,2‐diazaphosphochloride [(CH_2_)NDipp)_2_PCl. Reactions with strong electrophiles such as ^*i*^Pr_3_SiOTf or Ge(^Dipp^NacNac)OTf at room temperature were found to decompose to intractable, insoluble precipitates. [K(18‐crown‐6]**2** is also unreactive towards carbodiimides, which are known to readily undergo [2+2] cycloaddition reactions with PCO^−^ and AsCO^−^.[^12^, ^14^]

Nevertheless, protonation of [K(18‐crown‐6)]**2** with Brookhart's acid^[23]^ at −78 °C in THF (or Et_2_O) afforded a solution of (cyapho)dicyano‐methane HC(CN)_2_(CP), **3**, which is stable for more than 12 hours at −60 °C (Scheme 2). It is worth noting, however, that **3** decomposes within minutes on warming to −30 °C. No additional compounds could be observed by ^31^P NMR spectroscopy at this temperature, suggesting the formation of insoluble decomposition products. The existence of **3** was confirmed by multi‐element NMR spectroscopy at −60 °C. The ^31^P NMR spectrum of the reaction mixture revealed a doublet (^3^ *J*_P−H_=13.8 Hz) at −50.9 ppm. A doublet in the ^1^H NMR spectrum could also be observed with same coupling constant at 6.03 ppm. This resonance collapses to a singlet in the ^1^H{^31^P} NMR spectrum. Resonances for all three magentically inequivalent carbon atoms in **3** could be detected by ^13^C−^1^H 2D NMR spectroscopy. The central methine carbon was observed as a doublet at 23.92 ppm with ^2^ *J*_C−P_=22.7 Hz. The chemical shifts for the cyanide and cyaphide moieties were observed by ^13^C{^1^H} and HMBC NMR spectroscopy at 110.86 and 145.06 ppm, respectively. These values are in good agreement with computed chemical shifts (Table 1). These findings all are all consistent with the *in situ* generation of cyapho(dicyano)methane, and with protonation at the central carbon position. Indeed, calculations also suggest that cyapho(dicyano)methane is the most thermodynamically stable acid (by at least 5.0 kcal mol^−1^) when compared to the other isomers that could be formed on protonation of **2** (Figure S34). These data are in line with the values reported by Kornath for tricyanomethane.^[2]^

**Scheme 2:**
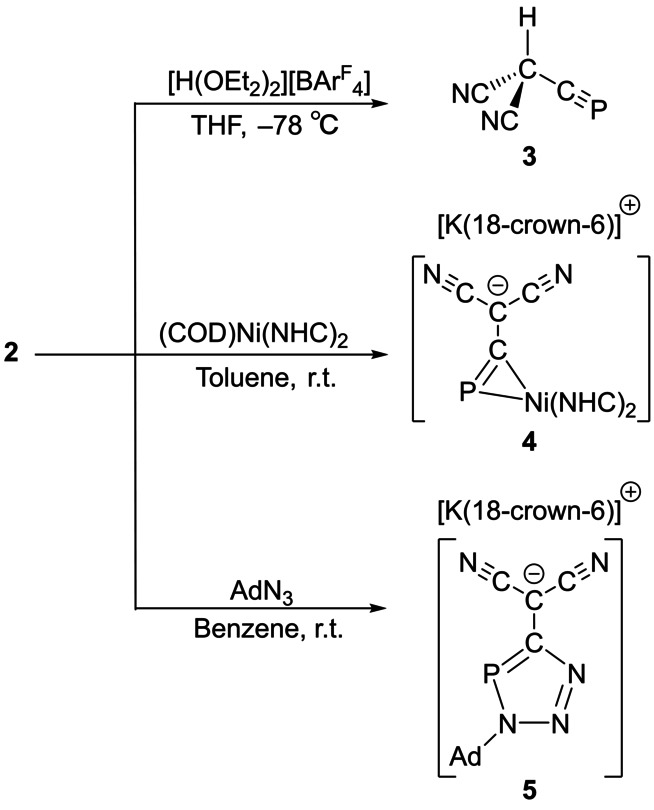
Reactivity of **2** leading to **3**–**5**. [K(18‐crown‐6)]**4** and [K(18‐crown‐6)]**5** were isolated in 40 and 49 % yield, respectively.

**Table 1:** NMR data for cyapho(dicyano)methane (**3**). Chemical shift values in ppm, coupling constants in Hz. (Calculated NMR shifts are referenced relative to ^*t*^BuCP [*δ*(^31^P)=−69.2 ppm; *δ*(^13^C *C*P moiety)=184.8 ppm].^[24]^

Nucleus	Experimental	Computed^[a]^
*δ*(^31^P) (^3^ *J*_P‐H_)	−50.1 (13.8) in d_8_‐THF −43.7 (13.7) in Et_2_O	−39.8
*δ*(^13^C) of cyaphide	145.06 in d_8_‐THF	142.3
*δ*(^13^C) of methine	23.92 in d_8_‐THF	15.2
*δ*(^13^C) of cyanide	110.86 in d_8_‐THF	103.5

[a] GIAO‐PBE1PBE/6‐311G(d,p)].

Having established that **2** is capable of reacting through the methanide carbon atom, we turned our attention to the reactivity of the cyaphide moiety. DFT calculations reveal that the lowest unoccupied molecular orbital (LUMO) of **2** has a significant contribution from an out‐of‐plane C−P π* interaction (Figure S30), which implies that reactions with the C≡P triple bond should be accessible. As phosphaalkynes are known to bind to transition metals complexes,^[25]^ we reasoned that reaction of **2** with an electron‐rich transition metal fragment should allow access to the cyaphide moiety through LUMO‐driven reactivity.^[26]^ Treatment of **2** with [Ni(COD)(^Me^IPr)_2_] (^Me^IPr=C[N^*i*^PrCMe]_2_) in toluene afforded complex **4**, which features a singlet in its ^31^P NMR spectrum at 45.4 ppm. The solid‐state molecular structure of [K(18‐crown‐6]**4**⋅1.5 tol was determined by single crystal X‐ray diffraction (Figure 4, top), which revealed a structure containing C−P−Ni three‐membered ring as expected. The bond length of C−P bond length (1.672(2) Å) is significantly elongated compared to that of **2** (1.553(2) Å), consistent with significant back‐donation from the nickel center to the C−P π* orbital. The cyapho(dicyano)‐methanide moiety in **4** still retains its planar geometry (Σ°: 359.8°), indicating that the molecule is still highly conjugated. Of note is that the P1−C1−C2 bond angle is now significantly bent (143.8(1)°) relative to that of **2** (179.6(2)°). This geometry is very similar to that observed for [Ni(IMes)(CO)(η^2^‐PC^*t*^Bu)] reported by Wolf in 2019,^[27]^ which has a C−P bond of 1.626(2) Å and a P−C−C bond angle of 146.1(2)°.

**Figure 4:**
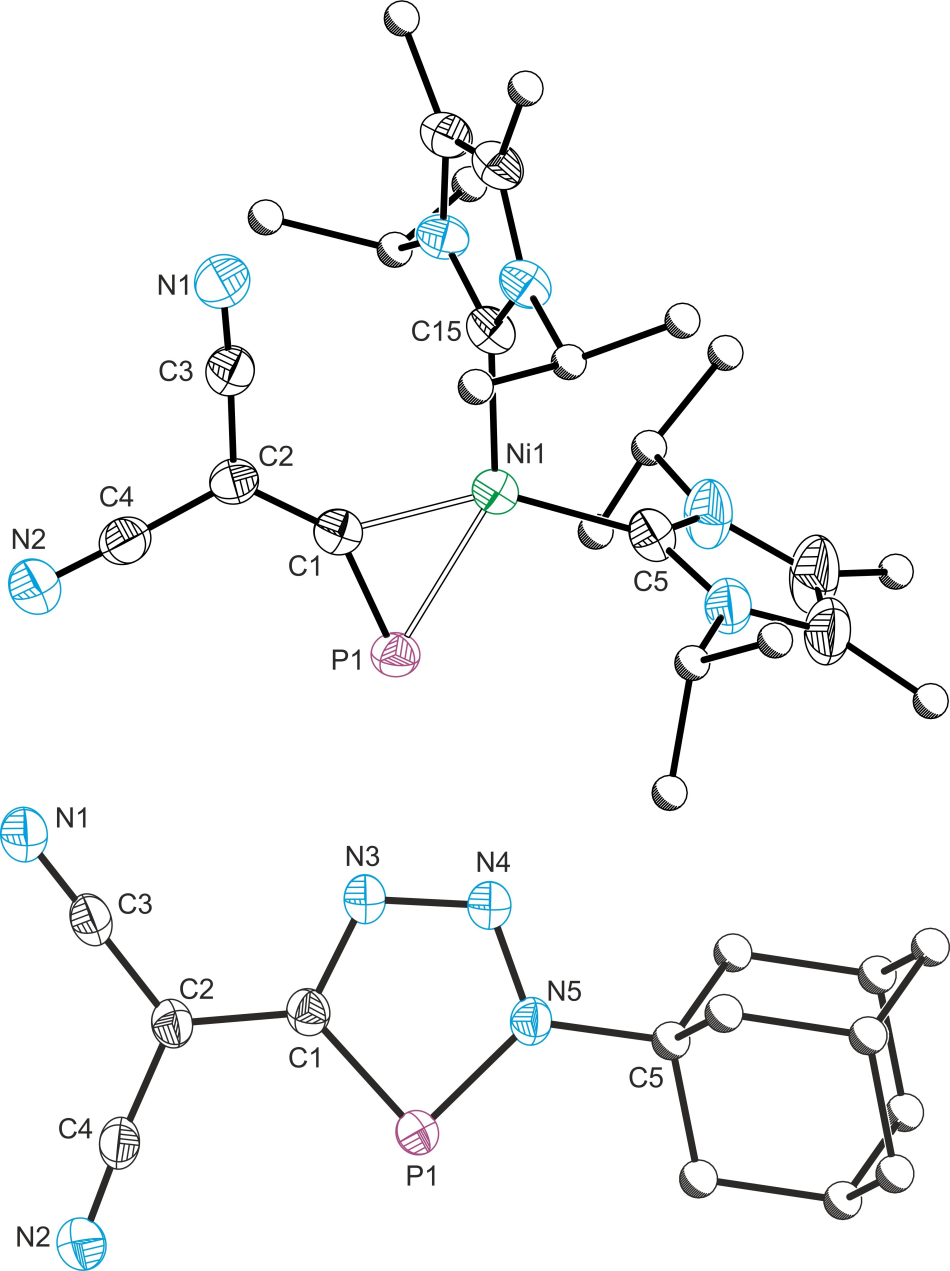
Single crystal X‐ray structures of the anionic components in [K(18‐crown‐6]**4**⋅1.5 tol (top) and [K(18‐crown‐6)]**5** (bottom). Anisotropic displacement ellipsoids pictured at 50 % probability. Carbon atoms of Me, ^*i*^Pr and Ad groups pictured as spheres of arbitrary radius. Selected bond lengths [Å] and angles [°] **4**: P1−C1, 1.672(2); C1−C2 1.416(2); C2−C3, 1.414(2), C2−C4, 1.418(2), C3−N1, 1.159(2), C4−N2, 1.157(2), P1−Ni1, 2.210(1); P1−C1, 1.672(2); C1−C2−C3, 123.6(2), C1−C2−C4, 123.4(2); C3−C2−C4, 112.8(2); C2−C1−P2, 143.8(1); Ni1−C1−P1, 76.8(1); C1−Ni1−P1, 47.4(1); Ni1−P1−C1 55.7(1). **5**: P1−C1, 1.740(2); C1−C2 1.442(2); C2−C3, 1.409(2), C2−C4, 1.403(2), C3−N1, 1.160(2), C4−N2, 1.158(2), C1−N3, 1.364(2); N3−N4, 1.316(2); N4−N5, 1.331(2); P1−N5, 1.706(1); C1−C2−C3, 122.3(2), C1−C2−C4, 118.4(2); C3−C2−C4, 119.3(2); C2−C1−P1, 126.3(1); C1−N3−N4, 113.1(2); N3−N4−N5, 112.1(1); N4−N5−P1 116.0(1); N5−P1−C1 86.1(1).

Prompted by the phosphaalkyne‐like nature of the bonding in **4**, we decided to explore other reactions of the C−P bond. Cyclisation reactions between azides and phosphaalkynes are known to yield triazaphospholes,^[28]^ however, to the best of our knowledge, no such reactivity has been reported for *anions* that contain a cyaphide moiety such as PCO^−^ or [N(CN)(CP)]^−^. Given the extensive delocalization present in **2**, it seemed viable that [3+2] cyclisation reactions might indeed take place with azides due to the relatively small degree of negative charge located on the cyaphide moiety (see Figure S32 for NPA analysis). Reaction of **2** with adamantyl azide revealed the formation of a new compound by ^31^P NMR spectroscopy with a singlet at 136.5 ppm. This is comparable to other known trizaphospholes such as 3,5‐di‐*tert*‐butyl‐1,2,3,4‐triazaphosphole which has a ^31^P chemical shift at 161.8 ppm.^[28c]^ The structure of the resulting product, [K(18‐crown‐6)]**5**, was confirmed by single crystal X‐ray diffraction (Figure 4, bottom). The most notable aspect of this structure is the fact that the triazaphosphole and dicyanomethanide moieties are co‐planar (mean deviation from plane 0.0156 Å), which is once again indicative of extensive delocalization of negative charge throughout the anion. As expected, on cyclisation the C−P bond elongates significant to 1.740(2) Å (cf. 1.553(2) Å for **2**).

In summary, we have developed a synthetic route to access a novel cyaphide‐containing anion, (cyapho)dicyano‐methanide [C(CN)_2_CP]^−^ (**2**), from the reaction of 1,1‐diethoxy‐2,2‐dicyanoethylene (EtO)_2_C=C(CN)_2_ with [Na(18‐crown‐6)][PH_2_]. An intermediate, [HP{C(OEt)=C(CN)_2_}]^−^, was isolated after the first Michael addition‐elimination step, and with the help of a non‐nucleophilic base (KHMDS), this intermediate could further be converted to the target anion. DFT calculations reveal that **2** possesses a highly delocalized π‐system and that the cyaphide moiety is just as (if not more) effective at negative charge stabilization than a nitrile group. The parent acid, (cyapho)dicyanomethane could also be synthesized and characterized at −60 °C by multi‐element NMR spectroscopic studies. Further reactivity studies revealed that the C≡P triple bond in **2** is chemically accessible by reaction with transition metal nucleophiles and azides. These findings suggest that, as with TCM, this novel anion may find use as a component in coordination polymers.

## Conflict of interest

The authors declare no conflict of interest.

## Supporting information

As a service to our authors and readers, this journal provides supporting information supplied by the authors. Such materials are peer reviewed and may be re‐organized for online delivery, but are not copy‐edited or typeset. Technical support issues arising from supporting information (other than missing files) should be addressed to the authors.

Supporting Information

Supporting Information

Supporting Information

## Data Availability

The data that support the findings of this study are available in the Supporting Information of this article.
