# Supplementary material for: Synthesis, Structure and Reactivity of a Cyapho(dicyano)methanide Salt
Source: Angew Chem Int Ed Engl. 2022 Aug 18;61(39):e202208921. doi: 10.1002/anie.202208921 (PMC9805078; doi:10.1002/anie.202208921)

checkCIF/PLATON report

You have not supplied any structure factors. As a result the full set of tests cannot be run.

THIS REPORT IS FOR GUIDANCE ONLY. IF USED AS PART OF A REVIEW PROCEDURE FOR PUBLICATION, IT SHOULD NOT REPLACE THE EXPERTISE OF AN EXPERIENCED CRYSTALLOGRAPHIC REFEREE.

No syntax errors found. CIF dictionary Interpreting this report

Datablock: CH122

Bond precision: C-C = 0.0027 A Wavelength=1.54184

Cell:	a=9.0861(3)	b=9.1743(3)	c=16.4208(4)
	alpha=87.976(2)	beta=82.419(2)	gamma=89.177(2)
Temperature:	150 K		

	Calculated	Reported
Volume	1355.94(7)	1355.94(7)
Space group	P -1	P -1
Hall group	-P 1	-P 1
Moiety formula	C44 H76 N4 Na2 O16 P2	C6 H6 N2 O P, C12, H24 O6 Na, C4 H8 O
Sum formula	C44 H76 N4 Na2 O16 P2	C22 H38 N2 Na O8 P
Mr	1025.01	512.50
Dx,g cm-3	1.255	1.255
Z	1	2
Mu (mm-1)	1.446	1.446
F000	548.0	548.0
F000'	550.49	
h,k,lmax	11,11,20	11,11,20
Nref	5686	5617
Tmin,Tmax	0.865,0.891	0.961,1.000
Tmin'	0.865	

Correction method= # Reported T Limits: Tmin=0.961 Tmax=1.000
AbsCorr = MULTI-SCAN

Data completeness= 0.988 Theta(max)= 76.160

R(reflections)= 0.0434(4694) wR2(reflections)= 0.1308(5617)

S = 1.064 Npar= 312

The following ALERTS were generated. Each ALERT has the format
test-name_ALERT_alert-type_alert-level.
Click on the hyperlinks for more details of the test.

Alert level C

```
PLAT042_ALERT_1_C Calc. and Reported MoietyFormula Strings  Differ     Please Check
```

Alert level G

```
PLAT045_ALERT_1_G Calculated and Reported Z Differ by a Factor ...      0.500 Check
PLAT154_ALERT_1_G The s.u.'s on the Cell Angles are Equal ..(Note)      0.002 Degree
PLAT230_ALERT_2_G Hirshfeld Test Diff for    C2        --C3       .       5.8 s.u.
PLAT720_ALERT_4_G Number of Unusual/Non-Standard Labels .........           8 Note
PLAT764_ALERT_4_G Overcomplete CIF Bond List Detected (Rep/Expd) .       1.11 Ratio
PLAT780_ALERT_1_G Coordinates do not Form a Properly Connected Set     Please Do !
```

0 **ALERT level A** = Most likely a serious problem - resolve or explain
0 **ALERT level B** = A potentially serious problem, consider carefully
1 **ALERT level C** = Check. Ensure it is not caused by an omission or oversight
6 **ALERT level G** = General information/check it is not something unexpected

4 ALERT type 1 CIF construction/syntax error, inconsistent or missing data
1 ALERT type 2 Indicator that the structure model may be wrong or deficient
0 ALERT type 3 Indicator that the structure quality may be low
2 ALERT type 4 Improvement, methodology, query or suggestion
0 ALERT type 5 Informative message, check

Datablock: CH138

Bond precision: C-C = 0.0030 A Wavelength=1.54184

Cell:	a=8.0042(5)	b=9.8316(4)	c=16.1020(8)
	alpha=95.380(4)	beta=100.530(4)	gamma=91.780(4)
Temperature:	150 K		

	Calculated	Reported
Volume	1238.80(11)	1238.80(11)
Space group	P -1	P -1
Hall group	-P 1	-P 1
Moiety formula	C20 H24 K N4 O6 P2, C20 H40 K O8	C4 N2 P, C12 H24 K O6, C4 H8 O
Sum formula	C40 H64 K2 N4 O14 P2	C20 H32 K N2 O7 P
Mr	965.09	482.54
Dx,g cm-3	1.294	1.294
Z	1	2
Mu (mm-1)	2.837	2.837
F000	512.0	512.0
F000'	514.85	
h,k,lmax	10,12,20	10,12,20
Nref	5672	5658
Tmin,Tmax	0.706,0.893	0.926,1.000
Tmin'	0.640	

Correction method= # Reported T Limits: Tmin=0.926 Tmax=1.000
AbsCorr = MULTI-SCAN

Data completeness= 0.998 Theta(max)= 89.757

R(reflections)= 0.0439(4274) wR2(reflections)= 0.1121(5658)

S = 1.040 Npar= 353

The following ALERTS were generated. Each ALERT has the format
test-name_ALERT_alert-type_alert-level.
Click on the hyperlinks for more details of the test.

Alert level C

PLAT042_ALERT_1_C Calc. and Reported MoietyFormula Strings Differ Please Check

---

Alert level G

```
PLAT003_ALERT_2_G Number of Uiso or Uij Restrained non-H Atoms ...         17 Report
PLAT045_ALERT_1_G Calculated and Reported Z Differ by a Factor ...      0.500 Check
PLAT154_ALERT_1_G The s.u.'s on the Cell Angles are Equal ..(Note)      0.004 Degree
PLAT171_ALERT_4_G The CIF-Embedded .res File Contains EADP Records          2 Report
PLAT177_ALERT_4_G The CIF-Embedded .res File Contains DELU Records          3 Report
PLAT178_ALERT_4_G The CIF-Embedded .res File Contains SIMU Records          3 Report
PLAT180_ALERT_4_G Check Cell Rounding: # of Values Ending with 0 =          4 Note
PLAT301_ALERT_3_G Main Residue  Disorder .............(Resd  1  )        55% Note
PLAT720_ALERT_4_G Number of Unusual/Non-Standard Labels ..........         20 Note
PLAT811_ALERT_5_G No ADDSYM Analysis: Too Many Excluded Atoms ....          ! Info
PLAT860_ALERT_3_G Number of Least-Squares Restraints ............         108 Note
```

PLAT941_ALERT_3_G Average HKL Measurement Multiplicity .... 3.9 Low

---

0 **ALERT level A** = Most likely a serious problem - resolve or explain
0 **ALERT level B** = A potentially serious problem, consider carefully
1 **ALERT level C** = Check. Ensure it is not caused by an omission or oversight
12 **ALERT level G** = General information/check it is not something unexpected

3 ALERT type 1 CIF construction/syntax error, inconsistent or missing data
1 ALERT type 2 Indicator that the structure model may be wrong or deficient
3 ALERT type 3 Indicator that the structure quality may be low
5 ALERT type 4 Improvement, methodology, query or suggestion
1 ALERT type 5 Informative message, check

Datablock: CH189

Bond precision: C-C = 0.0025 A Wavelength=1.54184

Cell:	a=11.1847(1)	b=33.9741(2)	c=13.9174(1)
	alpha=90	beta=91.247(1)	gamma=90
Temperature:	150 K		

	Calculated	Reported
Volume	5287.22(7)	5287.22(7)
Space group	P 21/n	P 21/n
Hall group	-P 2yn	-P 2yn
Moiety formula	2(C38 H64 K N6 Ni O6 P), 3(C7 H8)	C26 H40 N6 P Ni, C12 H24 K O6, 1.5(C7 H8)
Sum formula	C97 H152 K2 N12 Ni2 O12 P2	C48.50 H76 K N6 Ni O6 P
Mr	1935.83	967.93
Dx,g cm-3	1.216	1.216
Z	2	4
Mu (mm-1)	1.915	1.915
F000	2076.0	2076.0
F000'	2071.82	
h,k,lmax	14,42,17	13,42,17
Nref	11071	10987
Tmin,Tmax	0.871,0.926	0.703,1.000
Tmin'	0.656	

Correction method= # Reported T Limits: Tmin=0.703 Tmax=1.000
AbsCorr = MULTI-SCAN

Data completeness= 0.992 Theta(max)= 76.193

R(reflections)= 0.0316(9702)

wR2(reflections)= 0.0893(10987)

S = 1.031 Npar= 650

The following ALERTS were generated. Each ALERT has the format
test-name_ALERT_alert-type_alert-level.
Click on the hyperlinks for more details of the test.

Alert level B

```
PLAT230_ALERT_2_B Hirshfeld Test Diff for    P1       --C1        .         7.3 s.u.
```

Alert level C

```
PLAT042_ALERT_1_C Calc. and Reported MoietyFormula Strings  Differ     Please Check
PLAT220_ALERT_2_C NonSolvent   Resd 1  C   Ueq(max)/Ueq(min) Range        3.8 Ratio
PLAT223_ALERT_4_C Solv./Anion  Resd 2  H   Ueq(max)/Ueq(min) Range        4.8 Ratio
PLAT242_ALERT_2_C Low    'MainMol' Ueq as Compared to Neighbors of        C10 Check
PLAT250_ALERT_2_C Large U3/U1 Ratio for Average U(i,j) Tensor ....        3.7 Note
PLAT260_ALERT_2_C Large Average Ueq of Residue Including       C1R      0.109 Check
```

Alert level G

```
PLAT002_ALERT_2_G Number of Distance or Angle Restraints on AtSite         14 Note
PLAT045_ALERT_1_G Calculated and Reported Z Differ by a Factor ...      0.500 Check
PLAT142_ALERT_4_G s.u. on b - Axis Small or Missing .............     0.00020 Ang.
PLAT143_ALERT_4_G s.u. on c - Axis Small or Missing .............     0.00010 Ang.
PLAT175_ALERT_4_G The CIF-Embedded .res File Contains SAME Records          1 Report
PLAT230_ALERT_2_G Hirshfeld Test Diff for    C2       --C3        .         6.8 s.u.
PLAT230_ALERT_2_G Hirshfeld Test Diff for    C2       --C4        .         6.7 s.u.
PLAT232_ALERT_2_G Hirshfeld Test Diff (M-X)  Ni1      --P1        .         5.9 s.u.
PLAT232_ALERT_2_G Hirshfeld Test Diff (M-X)  Ni1      --C1        .         6.3 s.u.
PLAT232_ALERT_2_G Hirshfeld Test Diff (M-X)  Ni1      --C5        .         6.7 s.u.
PLAT232_ALERT_2_G Hirshfeld Test Diff (M-X)  Ni1      --C16       .         7.8 s.u.
PLAT300_ALERT_4_G Atom Site Occupancy of C1R       Constrained at         0.5 Check
PLAT300_ALERT_4_G Atom Site Occupancy of C2R       Constrained at         0.5 Check
PLAT300_ALERT_4_G Atom Site Occupancy of C3R       Constrained at         0.5 Check
PLAT300_ALERT_4_G Atom Site Occupancy of C4R       Constrained at         0.5 Check
PLAT300_ALERT_4_G Atom Site Occupancy of C5R       Constrained at         0.5 Check
PLAT300_ALERT_4_G Atom Site Occupancy of C6R       Constrained at         0.5 Check
PLAT300_ALERT_4_G Atom Site Occupancy of C7R       Constrained at         0.5 Check
PLAT300_ALERT_4_G Atom Site Occupancy of H3R       Constrained at         0.5 Check
PLAT300_ALERT_4_G Atom Site Occupancy of H4R       Constrained at         0.5 Check
PLAT300_ALERT_4_G Atom Site Occupancy of H5R       Constrained at         0.5 Check
PLAT300_ALERT_4_G Atom Site Occupancy of H6R       Constrained at         0.5 Check
PLAT300_ALERT_4_G Atom Site Occupancy of H1R1      Constrained at         0.5 Check
PLAT300_ALERT_4_G Atom Site Occupancy of H7R       Constrained at         0.5 Check
PLAT300_ALERT_4_G Atom Site Occupancy of H1R2      Constrained at         0.5 Check
PLAT300_ALERT_4_G Atom Site Occupancy of H1R3      Constrained at         0.5 Check
PLAT302_ALERT_4_G Anion/Solvent/Minor-Residue Disorder (Resd  2  )       100% Note
PLAT302_ALERT_4_G Anion/Solvent/Minor-Residue Disorder (Resd  3  )       100% Note
PLAT302_ALERT_4_G Anion/Solvent/Minor-Residue Disorder (Resd  4  )       100% Note
PLAT304_ALERT_4_G Non-Integer Number of Atoms in ..... (Resd  2  )       7.50 Check
PLAT304_ALERT_4_G Non-Integer Number of Atoms in ..... (Resd  3  )       8.20 Check
PLAT304_ALERT_4_G Non-Integer Number of Atoms in ..... (Resd  4  )       6.80 Check
```

```
PLAT343_ALERT_2_G Unusual sp?      Angle Range in Main Residue for         C1 Check
PLAT720_ALERT_4_G Number of Unusual/Non-Standard Labels ..........          9 Note
PLAT779_ALERT_4_G Suspect or Irrelevant (Bond) Angle(s) in CIF ...      41.16 Deg.
              N1   -C3   -K1      1_555   1_555   1_555 ......     #   15 Check
PLAT789_ALERT_4_G Atoms with Negative _atom_site_disorder_group  #         15 Check
PLAT860_ALERT_3_G Number of Least-Squares Restraints ............         15 Note
```

---

   0 **ALERT level A** = Most likely a serious problem - resolve or explain
   1 **ALERT level B** = A potentially serious problem, consider carefully
   6 **ALERT level C** = Check. Ensure it is not caused by an omission or oversight
  37 **ALERT level G** = General information/check it is not something unexpected

   2 ALERT type 1 CIF construction/syntax error, inconsistent or missing data
  13 ALERT type 2 Indicator that the structure model may be wrong or deficient
   1 ALERT type 3 Indicator that the structure quality may be low
  28 ALERT type 4 Improvement, methodology, query or suggestion
   0 ALERT type 5 Informative message, check

Datablock: CH022

Bond precision: C-C = 0.0020 A   Wavelength=1.54184

Cell:	a=10.1564(3)	b=10.7107(3)	c=15.3083(4)
	alpha=75.303(2)	beta=82.672(2)	gamma=65.466(3)
Temperature:	150 K		

	Calculated	Reported
Volume	1464.89(8)	1464.89(8)
Space group	P -1	P -1
Hall group	-P 1	-P 1
Moiety formula	C26 H39 K N5 O6 P	C14 H15 N5 P, C12 H24 K O6
Sum formula	C26 H39 K N5 O6 P	C26 H39 K N5 O6 P
Mr	587.69	587.69
Dx,g cm-3	1.332	1.332
Z	2	2
Mu (mm-1)	2.503	2.503
F000	624.0	624.0
F000’	627.11	
h,k,lmax	12,13,19	12,13,19
Nref	6144	6082
Tmin,Tmax	0.740,0.779	0.945,1.000
Tmin’	0.671	

Correction method= # Reported T Limits: Tmin=0.945 Tmax=1.000
AbsCorr = MULTI-SCAN

Data completeness= 0.990 Theta(max)= 76.269

R(reflections)= 0.0300(5413) wR2(reflections)= 0.0786(6082)

S = 1.037 Npar= 439

The following ALERTS were generated. Each ALERT has the format
test-name_ALERT_alert-type_alert-level.
Click on the hyperlinks for more details of the test.

Alert level C

```
PLAT042_ALERT_1_C Calc. and Reported MoietyFormula Strings  Differ     Please Check
PLAT215_ALERT_3_C Disordered C5A       has ADP max/min Ratio .....        3.4 Note
PLAT215_ALERT_3_C Disordered C204      has ADP max/min Ratio .....        3.3 Note
PLAT220_ALERT_2_C NonSolvent   Resd 1  C   Ueq(max)/Ueq(min) Range        5.0 Ratio
PLAT222_ALERT_3_C NonSolvent Resd 1  H   Uiso(max)/Uiso(min) Range        4.4 Ratio
PLAT329_ALERT_4_C Carbon Atom Hybridisation Unclear for ..........       C201 Check
```

Alert level G

```
PLAT002_ALERT_2_G Number of Distance or Angle Restraints on AtSite         18 Note
PLAT003_ALERT_2_G Number of Uiso or Uij Restrained non-H Atoms ...         18 Report
PLAT004_ALERT_5_G Polymeric Structure Found with Maximum Dimension          1 Info
PLAT175_ALERT_4_G The CIF-Embedded .res File Contains SAME Records          1 Report
PLAT177_ALERT_4_G The CIF-Embedded .res File Contains DELU Records          3 Report
PLAT178_ALERT_4_G The CIF-Embedded .res File Contains SIMU Records          3 Report
PLAT230_ALERT_2_G Hirshfeld Test Diff for    C2       --C3       .        5.9 s.u.
PLAT230_ALERT_2_G Hirshfeld Test Diff for    C2       --C4       .        6.2 s.u.
PLAT300_ALERT_4_G Atom Site Occupancy of K2          Constrained at       0.5 Check
PLAT300_ALERT_4_G Atom Site Occupancy of O1A         Constrained at       0.5 Check
PLAT300_ALERT_4_G Atom Site Occupancy of O2A         Constrained at       0.5 Check
PLAT300_ALERT_4_G Atom Site Occupancy of O3A         Constrained at       0.5 Check
PLAT300_ALERT_4_G Atom Site Occupancy of O201        Constrained at       0.5 Check
PLAT300_ALERT_4_G Atom Site Occupancy of O202        Constrained at       0.5 Check
PLAT300_ALERT_4_G Atom Site Occupancy of O203        Constrained at       0.5 Check
PLAT300_ALERT_4_G Atom Site Occupancy of C1A         Constrained at       0.5 Check
PLAT300_ALERT_4_G Atom Site Occupancy of C2A         Constrained at       0.5 Check
PLAT300_ALERT_4_G Atom Site Occupancy of C3A         Constrained at       0.5 Check
PLAT300_ALERT_4_G Atom Site Occupancy of C4A         Constrained at       0.5 Check
PLAT300_ALERT_4_G Atom Site Occupancy of C5A         Constrained at       0.5 Check
PLAT300_ALERT_4_G Atom Site Occupancy of C6A         Constrained at       0.5 Check
PLAT300_ALERT_4_G Atom Site Occupancy of C201        Constrained at       0.5 Check
PLAT300_ALERT_4_G Atom Site Occupancy of C202        Constrained at       0.5 Check
PLAT300_ALERT_4_G Atom Site Occupancy of C203        Constrained at       0.5 Check
PLAT300_ALERT_4_G Atom Site Occupancy of C204        Constrained at       0.5 Check
PLAT300_ALERT_4_G Atom Site Occupancy of C205        Constrained at       0.5 Check
PLAT300_ALERT_4_G Atom Site Occupancy of C206        Constrained at       0.5 Check
PLAT300_ALERT_4_G Atom Site Occupancy of H1A1        Constrained at       0.5 Check
PLAT300_ALERT_4_G Atom Site Occupancy of H1A2        Constrained at       0.5 Check
PLAT300_ALERT_4_G Atom Site Occupancy of H2A1        Constrained at       0.5 Check
PLAT300_ALERT_4_G Atom Site Occupancy of H2A2        Constrained at       0.5 Check
PLAT300_ALERT_4_G Atom Site Occupancy of H3A1        Constrained at       0.5 Check
PLAT300_ALERT_4_G Atom Site Occupancy of H3A2        Constrained at       0.5 Check
```

```
PLAT300_ALERT_4_G Atom Site Occupancy of H4A1       Constrained at       0.5 Check
PLAT300_ALERT_4_G Atom Site Occupancy of H4A2       Constrained at       0.5 Check
PLAT300_ALERT_4_G Atom Site Occupancy of H5A1       Constrained at       0.5 Check
PLAT300_ALERT_4_G Atom Site Occupancy of H5A2       Constrained at       0.5 Check
PLAT300_ALERT_4_G Atom Site Occupancy of H6A1       Constrained at       0.5 Check
PLAT300_ALERT_4_G Atom Site Occupancy of H6A2       Constrained at       0.5 Check
PLAT300_ALERT_4_G Atom Site Occupancy of H20A       Constrained at       0.5 Check
PLAT300_ALERT_4_G Atom Site Occupancy of H20B       Constrained at       0.5 Check
PLAT300_ALERT_4_G Atom Site Occupancy of H20C       Constrained at       0.5 Check
PLAT300_ALERT_4_G Atom Site Occupancy of H20D       Constrained at       0.5 Check
PLAT300_ALERT_4_G Atom Site Occupancy of H20E       Constrained at       0.5 Check
PLAT300_ALERT_4_G Atom Site Occupancy of H20F       Constrained at       0.5 Check
PLAT300_ALERT_4_G Atom Site Occupancy of H20G       Constrained at       0.5 Check
PLAT300_ALERT_4_G Atom Site Occupancy of H20H       Constrained at       0.5 Check
PLAT300_ALERT_4_G Atom Site Occupancy of H20I       Constrained at       0.5 Check
PLAT300_ALERT_4_G Atom Site Occupancy of H20J       Constrained at       0.5 Check
PLAT300_ALERT_4_G Atom Site Occupancy of H20K       Constrained at       0.5 Check
PLAT300_ALERT_4_G Atom Site Occupancy of H20L       Constrained at       0.5 Check
PLAT301_ALERT_3_G Main Residue  Disorder .............(Resd  1  )        24% Note
PLAT720_ALERT_4_G Number of Unusual/Non-Standard Labels ..........        12 Note
PLAT779_ALERT_4_G Suspect or Irrelevant (Bond) Angle(s) in CIF ...     27.18 Deg.
              K2   -O201 -K2      1_555   1_555   2_675 ......     #  201 Check
PLAT779_ALERT_4_G Suspect or Irrelevant (Bond) Angle(s) in CIF ...     24.01 Deg.
              K2   -O202 -K2      1_555   1_555   2_675 ......     #  207 Check
PLAT779_ALERT_4_G Suspect or Irrelevant (Bond) Angle(s) in CIF ...     22.73 Deg.
              K2   -O203 -K2      1_555   1_555   2_675 ......     #  213 Check
PLAT779_ALERT_4_G Suspect or Irrelevant (Bond) Angle(s) in CIF ...     25.14 Deg.
              K2   -O1A  -K2      1_555   1_555   2_675 ......     #  265 Check
PLAT779_ALERT_4_G Suspect or Irrelevant (Bond) Angle(s) in CIF ...     24.67 Deg.
              K2   -O2A  -K2      1_555   1_555   2_675 ......     #  269 Check
PLAT779_ALERT_4_G Suspect or Irrelevant (Bond) Angle(s) in CIF ...     24.80 Deg.
              K2   -O3A  -K2      1_555   1_555   2_675 ......     #  273 Check
PLAT779_ALERT_4_G Suspect or Irrelevant (Bond) Angle(s) in CIF ...     20.72 Deg.
              K2   -C6A  -K2      1_555   1_555   2_675 ......     #  314 Check
PLAT804_ALERT_5_G Number of ARU-Code Packing Problem(s) in PLATON          2 Info
PLAT860_ALERT_3_G Number of Least-Squares Restraints ............        149 Note
```

---

0 **ALERT level A** = Most likely a serious problem - resolve or explain
0 **ALERT level B** = A potentially serious problem, consider carefully
6 **ALERT level C** = Check. Ensure it is not caused by an omission or oversight
62 **ALERT level G** = General information/check it is not something unexpected

1 ALERT type 1 CIF construction/syntax error, inconsistent or missing data
5 ALERT type 2 Indicator that the structure model may be wrong or deficient
5 ALERT type 3 Indicator that the structure quality may be low
55 ALERT type 4 Improvement, methodology, query or suggestion
2 ALERT type 5 Informative message, check

It is advisable to attempt to resolve as many as possible of the alerts in all categories. Often the minor alerts point to easily fixed oversights, errors and omissions in your CIF or refinement strategy, so attention to these fine details can be worthwhile. In order to resolve some of the more serious problems it may be necessary to carry out additional measurements or structure refinements. However, the purpose of your study may justify the reported deviations and the more serious of these should normally be commented upon in the discussion or experimental section of a paper or in the "special_details" fields of the CIF. checkCIF was carefully designed to identify outliers and unusual parameters, but every test has its limitations and alerts that are not important in a particular case may appear. Conversely, the absence of alerts does not guarantee there are no aspects of the results needing attention. It is up to the individual to critically assess their own results and, if necessary, seek expert advice.

Publication of your CIF in IUCr journals

A basic structural check has been run on your CIF. These basic checks will be run on all CIFs submitted for publication in IUCr journals (*Acta Crystallographica*, *Journal of Applied Crystallography*, *Journal of Synchrotron Radiation*); however, if you intend to submit to *Acta Crystallographica Section C* or *E* or *IUCrData*, you should make sure that full publication checks are run on the final version of your CIF prior to submission.

Publication of your CIF in other journals

Please refer to the *Notes for Authors* of the relevant journal for any special instructions relating to CIF submission.

PLATON version of 18/05/2022; check.def file version of 17/05/2022

Datablock CH122 - ellipsoid plot

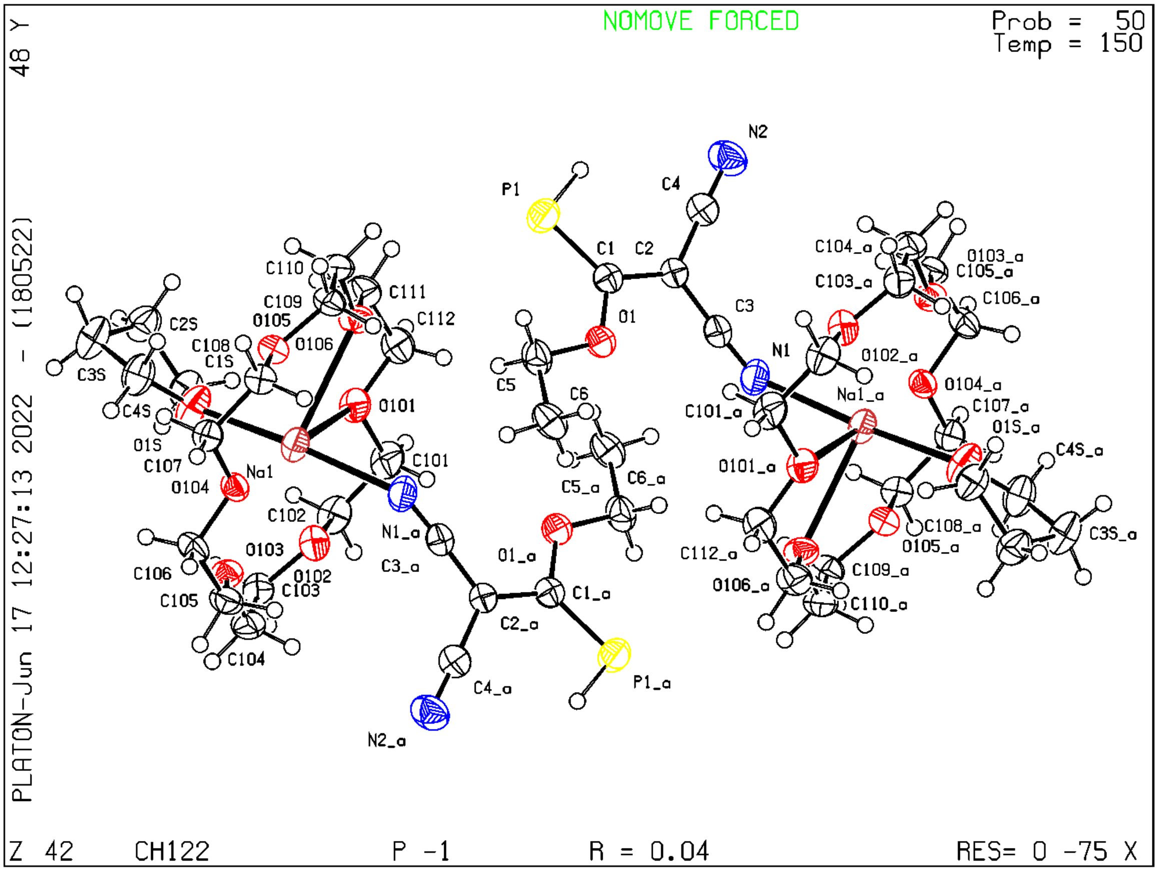

Datablock CH138 - ellipsoid plot

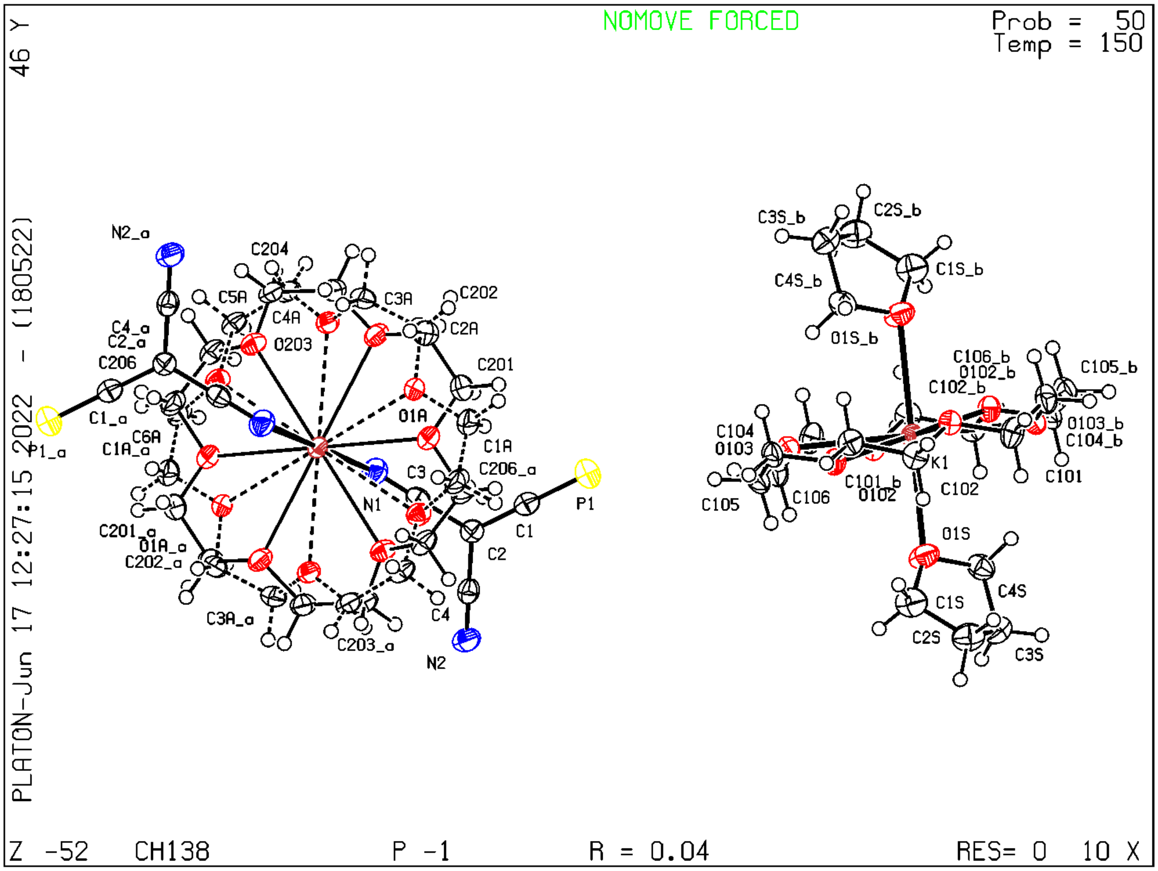

Datablock CH189 - ellipsoid plot

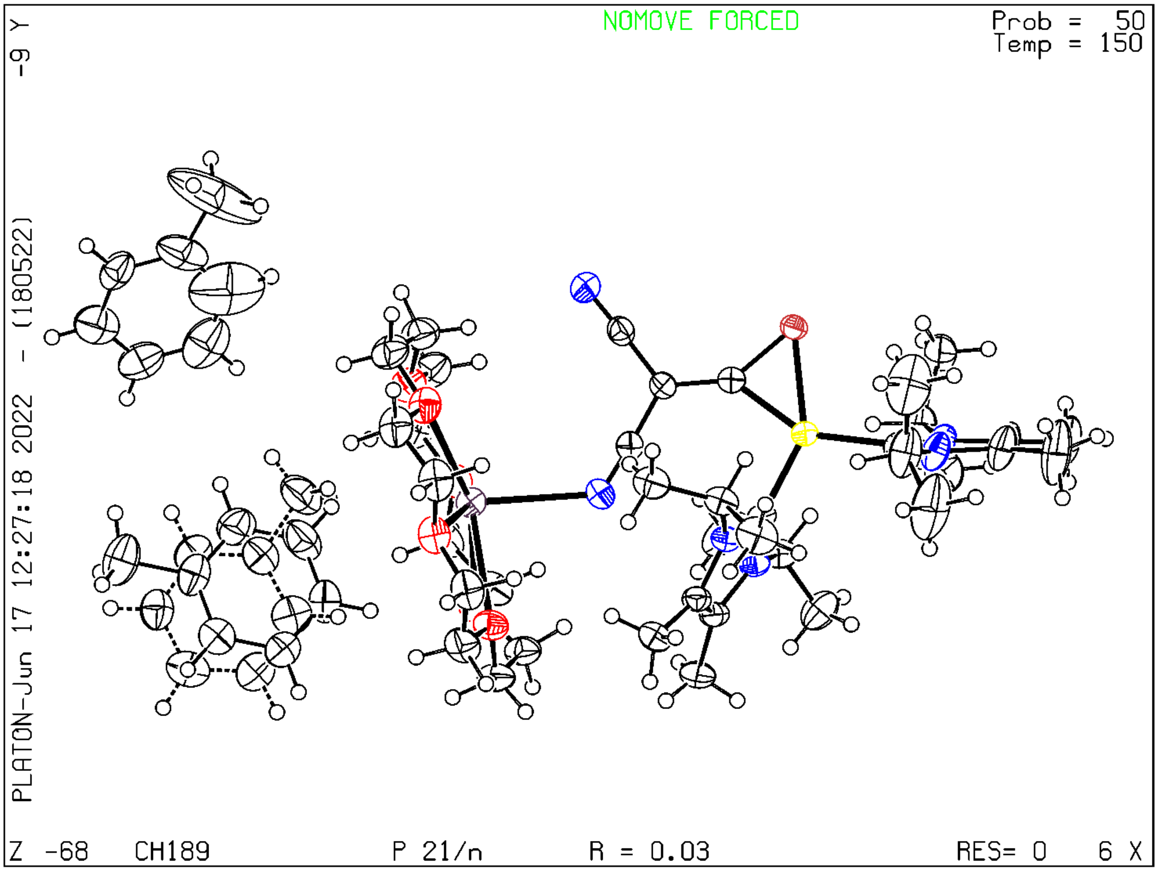

Datablock CH022 - ellipsoid plot

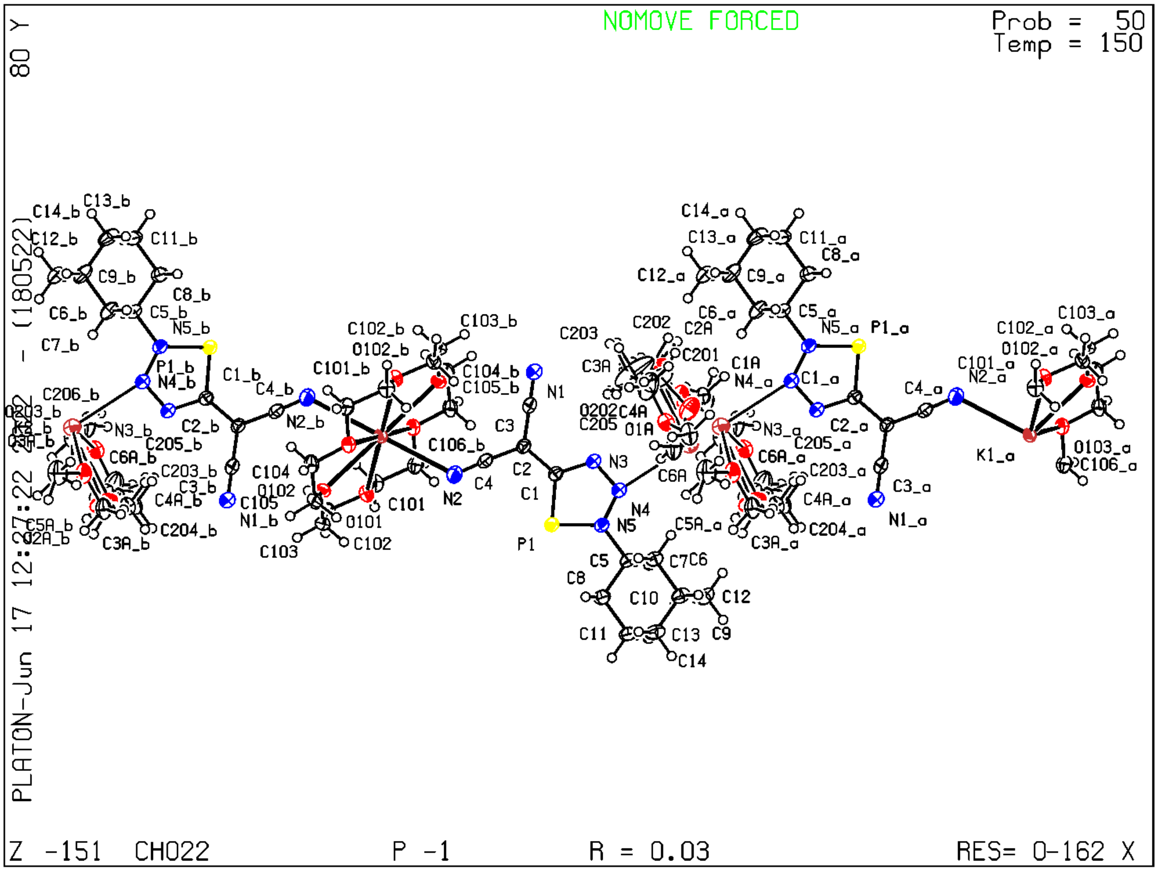

Supplement: Supplementary file 1 — Supporting Information [file ANIE-61-0-s003.pdf]
