# Supplementary material for: Synthesis, Structure and Reactivity of a Cyapho(dicyano)methanide Salt
Source: Angew Chem Int Ed Engl. 2022 Aug 18;61(39):e202208921. doi: 10.1002/anie.202208921 (PMC9805078; doi:10.1002/anie.202208921)
Supplement: Supplementary file 3 — Supporting Information [file ANIE-61-0-s001.pdf]

Angewandte Chemie
Eine Zeitschrift der Gesellschaft Deutscher Chemiker

Supporting Information

Synthesis, Structure and Reactivity of a Cyapho(dicyano)methanide Salt

*C. Hu, J. M. Goicoechea**

Supporting Information

Contents

1. Experimental details 2

2. IR and NMR spectra 8

3. X-ray Crystallographic Data 20

4. Computational Details 22

5. References 30

1. Experimental details

1.1. General Synthetic Methods

All reactions and product manipulations were carried out under an inert atmosphere of argon or dinitrogen using standard Schlenk-line or glovebox techniques (MBraun UNIlab glovebox maintained at < 0.1 ppm H_2O and < 0.1 ppm O_2). Hexane (hex; Sigma Aldrich HPLC grade), pentane (pent; Sigma Aldrich HPLC grade), and toluene (tol; Sigma Aldrich HPLC grade) were purified using an MBraun SPS-800 solvent system. Tetrahydrofuran (THF; Sigma Aldrich, ≥99.9%) and d_8-THF (Eurisotop, >99.5%) were distilled over a sodium metal/benzophenone mixture. C_6D_6 (Sigma Aldrich, 99.5%) was degassed prior to use. All dry solvents were stored under argon in airtight ampoules over activated 3 Å molecular sieves. Potassium hexamethyldisilazide (KHMDS, Sigma Aldrich, 97%), and 1-azidoadamantane (Sigma-Aldrich 97%) were stored in the glovebox and used as received. [Na(18-crown-6)][PH_2], 1,1-$(EtO)_2C{=}C(CN)_2$, [Ni(COD)(^{Me}IPr)$_2$] and Brookhart's acid [$H(OEt_2)]_2$][BAr^F_4] (BAr^F_4 = $B\{C_6H_3(CF_3)_2\}_4$) were prepared as described in the literature.[1–4]

Additional characterization techniques: NMR spectra were acquired on Bruker AVIII 400 MHz, Bruker AVIII HD 500 MHz (w/ detect ^{13}C cryoprobe), and Bruker NEO 600 MHz (w/ broadband helium cryoprobe) spectrometers. ^1H and ^{13}C NMR spectra were referenced to the most downfield protio-solvent resonance (^1H NMR C_6D_6: δ = 7.16 ppm; ^{13}C NMR C_6D_6: δ = 128.06 ppm; ^{13}C NMR d_8-THF = 67.57 ppm). ^1H NMR spectra in d_8-THF were referenced to the resonance at 1.73 ppm due to overlap of the most downfield resonance with the resonance arising from 18-crown-6. ^{31}P NMR spectra were externally referenced to an 85% solution of H_3PO_4 in H_2O. IR data were obtained by using attenuated total reflectance (ATR) method, spectra were recorded on a Thermo Scientific iS5 FTIR spectrometer in transmittance mode. Elemental analyses were carried out by Elemental Microanalyses Ltd. (Devon, U.K.). Samples (approx. 5 mg) were submitted in vacuum sealed Pyrex ampoules.

1.2. Synthesis of [Na(18-crown-6)]1

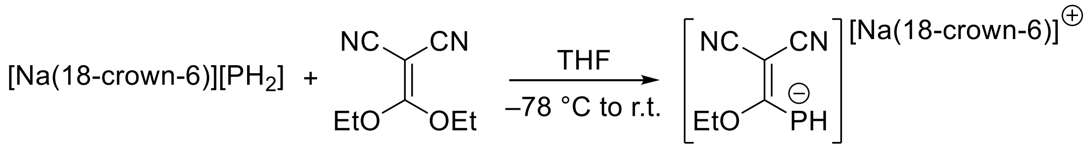

Figure S1. Synthesis of [Na(18-crown-6)]**1**.

A THF solution (3 mL) of 1,1-diethoxy-2,2-dicyanoethylene, $(EtO)_2C{=}C(CN)_2$, (150 mg, 0.90 mmol, 1.00 eq.) was added dropwise to a stirring THF solution (10 mL) of [Na(18-crown-6)][PH_2] (289 mg, 0.90 mmol, 1.00 eq.) at –78 °C. The reaction was allowed to slowly warm to room temperature overnight. All volatiles were removed *in vacuo*, and the resulting yellow oil was re-dissolved in minimal amount of THF (5 ml) and layered with pentane (THF:pentane = 1:1) at room temperature. Yellow, needle-like crystals were collected after 1 day and these were washed with toluene (2 × 2 mL) and pentane (2 × 2 mL) and dried under a dynamic vacuum to yield [Na(18-crown-6)]**1** (250 mg, 0.57 mmol; 63% yield). Single crystals of [Na(18-crown-6)]**1**·THF suitable for X-ray diffraction were obtained by slow diffusion of pentane into a concentrated THF solution of the product at –35°C for 3 days.

Anal. Calcd. for $C_{18}H_{30}N_2NaO_7P$ (M.W. 440.38 g mol^{-1}): C, 49.09; H, 6.87; N, 6.36; Found: C 48.98; H, 6.82; N, 6.60.

^{1}H NMR (600 MHz, d$_8$-THF): δ (ppm) 1.27 (t, $^3J_{H\text{–}H}$ = 7.0 Hz, 3H; CH_3), 3.14 (d, $^1J_{P\text{–}H}$ = 166.5 Hz, 1H; PH), 3.62 (s, 24H; 18-crown-6), 4.11 (dq, $^4J_{P\text{–}H}$ = 2.6 Hz, $^3J_{H\text{–}H}$ = 7.0 Hz, 2H; CH_2).

^{13}C{^{1}H} NMR (151 MHz, d$_8$-THF): δ (ppm) 15.00 (s; CH$_3$), 47.20 (d, $^2J_{P\text{–}C}$ = 11.9 Hz; (CN)$_2C$), 65.75 (d, $^3J_{P\text{–}C}$ = 37.1 Hz; OCH$_2$), 70.54 (s; 18-crown-6), 120.65 (br; CN), 124.86 (br; CN), 215.61 (d, $^1J_{P\text{–}C}$ = 62.0 Hz; C(PH)).

^{31}P NMR (162 MHz, d$_8$-THF): δ (ppm) −66.3 (d, $^1J_{P\text{–}H}$ = 166.5 Hz).

^{31}P{^{1}H} NMR (162 MHz, d$_8$-THF): δ (ppm) −66.7 (s).

1.3. Synthesis of [K(18-crown-6)]2

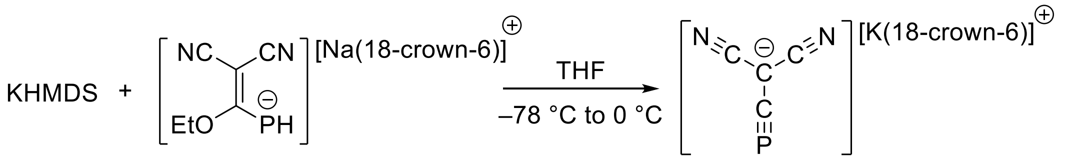

Figure S2. Synthesis of [K(18-crown-6)]**2**.

A THF solution (3 mL) of KHMDS (67.8 mg, 0.34 mmol, 1.00 eq.) was slowly added to a THF solution (10 mL) of [Na(18-crown-6)]**1** (150 mg, 0.34 mmol, 1.00 eq.) at –78 °C. After stirring for 3 hours at –78 °C, the solution was warmed to 0 °C and left stirring at this temperature for 2 days. The reaction was monitored by ^{31}P NMR spectroscopy until all of the starting material was completed consumed. The solution was filtered to remove NaOEt, concentrated to half of the original volume under vacuum at –35 °C, and layered with cold pentane. [Note: Concentration of this solution at temperatures above –30 °C gives rise to decomposition]. The THF/pentane mixture was shaken vigorously at –35 °C to form a brown precipitate, and the solvent removed by filtration. The remaining brown powder was dried *in vacuo* to yield [K(18-crown-6)]**2** (90 mg, 0.22 mmol; yield: 64%). In the solid state, the product can be stored at room temperature for over a month without decomposition. Crystals of [K(18-crown-6)]**2**·THF were obtained by slow diffusion of pentane into a THF solution at –35 °C over 3 days.

Anal. Calcd. for $C_{16}H_{24}KN_2O_6P$ (M.W. 410.42 g mol^{-1}): C, 46.82; H, 5.89; N, 6.83; Found: C 47.54; H, 6.04; N, 6.61.

^{1}H NMR (500 MHz, d_8-THF): δ (ppm) 3.62 (s, 18-crown-6).

^{13}C{^{1}H} NMR (125 MHz, d_8-THF): δ (ppm) 34.94 (d, $^{2}J_{P\text{–}C}$=19.3 Hz; *C*(CN)$_2$), 70.91 (s, 18-crown-6), 122.59 (d, $^{3}J_{P\text{–}C}$ = 4.8 Hz; *C*N), 171.00 (d, $^{1}J_{P\text{–}C}$ = 27.5 Hz; *C*P).

^{31}P NMR (162 MHz, d_8-THF): δ (ppm) 27.1 (s).

FTIP (ATR): ν(cm^{-1}) 1554.6 (vs, PCC stretch), 2151.7 (vs, NCC_{asymm} stretch), 2183.1 (vs, NCC_{symm} stretch).

1.4. Synthesis of 3

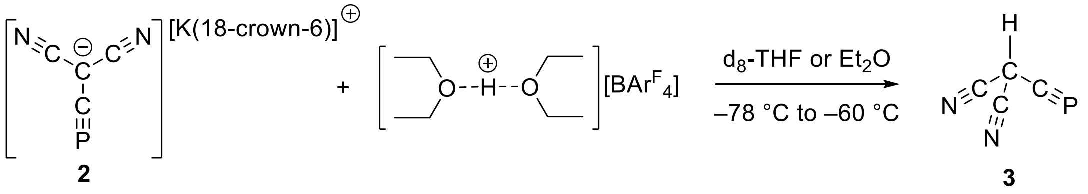

Figure S3. Synthesis of **3**.

[K(18-crown-6)]**2** (5 mg, 0.012 mmol, 1.00 eq.) and Brookhart's acid (12.3 mg, 0.012 mmol, 1.00 eq.) were weighed into an NMR tube equipped with an air-tight tap inside a glovebox. 0.4 ml of d_8-THF (or Et_2O) were vacuum transferred into the NMR tube and the solvent was allowed to thaw in an acetone-dry ice bath. A Bruker AVIII HD 500 MHz (w/ detect ^{13}C cryoprobe) spectrometer was pre-cooled to –60 °C before the reaction mixture was inserted into the instrument. All NMR data were recorded at this temperature. Based on the integration of ^{1}H NMR spectrum, around 40% conversion was achieved at –60 °C.

^{1}H NMR (500 MHz, d_8-THF, –60 °C): δ (ppm) 3.56 (s; 18-crown-6), 6.03 (d, $^{3}J_{P\text{–}H}$ = 13.8 Hz, 0.4H; *H*C(CN)$_2$(CP)), 1.10 (t, $^{3}J_{H\text{–}H}$ = 6.4 Hz, 12H; C*H*$_3$ Et_2O), 3.35 (q, $^{3}J_{H\text{–}H}$ = 6.4 Hz, 8H; C*H*$_2$ Et_2O), 7.70 (s, 4H; Ar*H* BAr^F_4), 7.86 (s, 8H; Ar*H* BAr^F_4), 13.10 (s broad, 0.6 H; H^+).

$^{13}C\{^{1}H\}$ NMR (125 MHz, d_8-THF, –60 °C): δ (ppm) 23.92 (d, $^{2}J_{P\text{–}C}$ = 22.7 Hz; H*C*(CN)$_2$(CP)), 70.80 (s, 18-crown-6) 110.86 (s; *C*N), 145.06 (*C*P), 15.73 (s; *C*H$_3$ Et_2O), 66.46 (s; *C*H$_2$ Et_2O), 118.23 (s; Ar*C* BAr^F_4), 125.21 (q, $^{1}J_{C\text{–}F}$ = 273.0 Hz; *C*F$_3$ BAr^F_4), 129.72 (q, $^{2}J_{C\text{–}F}$ = 31.8 Hz; Ar*C* BAr^F_4), 135.16 (s; Ar*C* BAr^F_4), 162.75 (q, $^{1}J_{C\text{–}B}$ = 49.5 Hz; Ar*C* BAr^F_4).

^{31}P NMR (202 MHz, d_8-THF, –60 °C): δ (ppm) −50.9 (d, $^{3}J_{P\text{–}H}$=13.8 Hz).

$^{31}P\{^{1}H\}$ NMR (202 MHz, d_8-THF, –60 °C): δ (ppm) −50.1 (s).

^{31}P NMR (202 MHz, Et_2O, –60 °C): δ (ppm) −43.7 (d, $^{3}J_{P\text{–}H}$=13.7 Hz).

$^{31}P\{^{1}H\}$ NMR (202 MHz, Et_2O, –60 °C): δ (ppm) −43.6 (s).

1.5. Synthesis of [K(18-crown-6)]4

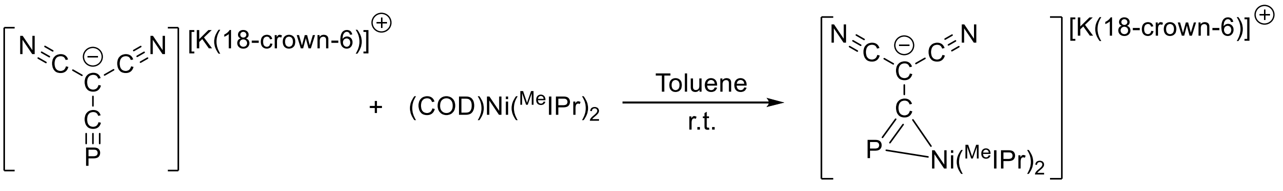

Figure S4. Synthesis of [K(18-crown-6)]**4**

A toluene solution (0.6 mL) of [Ni(COD)(^{Me}IPr)$_2$] (12.8 mg, 0.024mmol, 1.00 eq.) was slowly added to a toluene solution (0.6 mL) of [K(18-crown-6)]**2** (10 mg, 0.024mmol, 1.00 eq.). The mixture was stirred for 10 minutes before filtering. The resulting dark-brown solution was layered with pentane at −35 °C. Orange crystals were collected after 3 days and washed with pentane (2 × 1 mL), then dried *in vacuo* to afford [K(18-crown-6)]**4** (8 mg, 0.01 mmol; yield: 40%). Single crystal of [K(18-crown-6)]**4**·1.5tol suitable for X-ray diffraction were obtained by slow diffusion of pentane into a concentrated toluene/THF solution of the product at room temperature overnight.

Anal. Calcd for $C_{38}H_{64}KN_6NiO_6P$·0.5tol (M.W. 875.73 g mol^{-1}): C, 56.91; H, 7.83; N, 9.60; Found: C 56.30; H, 8.21; N, 9.28.

^{1}H NMR (600 MHz, C_6D_6): δ (ppm) 1.28 (d, $^3J_{H\text{–}H}$ = 7.1 Hz, 6H; CH(C*H*$_3$)$_2$), 1.32 (d broad, $^3J_{H\text{–}H}$ = 7.0 Hz, 12H, CH(C*H*$_3$)$_2$), 1.91 (s, 6H; C*H*$_3$), 2.04 (s, 6H; C*H*$_3$), 3.31 (s, 24H; 18-crown-6), 6.07 (sept, $^3J_{H\text{–}H}$ = 7.0 Hz, 2H; C*H*(CH$_3$)$_2$), 6.21 (sept, $^3J_{H\text{–}H}$ = 7.0 Hz, 2H; C*H*(CH$_3$)$_2$).

^{13}C{^{1}H} NMR (151 MHz, C_6D_6): δ (ppm) 10.58 (s; *C*H$_3$), 10.86 (s; *C*H$_3$), 22.06 (s; CH(*C*H$_3$)$_2$), 22.42 (s; CH(*C*H$_3$)$_2$), 22.51 (s; CH(*C*H$_3$)$_2$), 42.68 (s; *C*(CN)$_2$(CP)), 52.10 (s; *C*H(CH$_3$)$_2$), 52.21 (s; *C*H(CH$_3$)$_2$), 70.37 (s; 18-crown-6), 123.27 (s; *C*(CH$_3$)), 124.01 (s; *C*(CH$_3$)), 132.54 (s; *C*N), 201.16 (s; carbene *C*), 203.60 (s; carbene *C*), 219.20 (d, $^1J_{P\text{–}C}$ = 88.3 Hz; *C*P).

^{31}P NMR (162 MHz, C_6D_6): δ (ppm) 45.4 (s).

1.6. Synthesis of [K(18-crown-6)]5

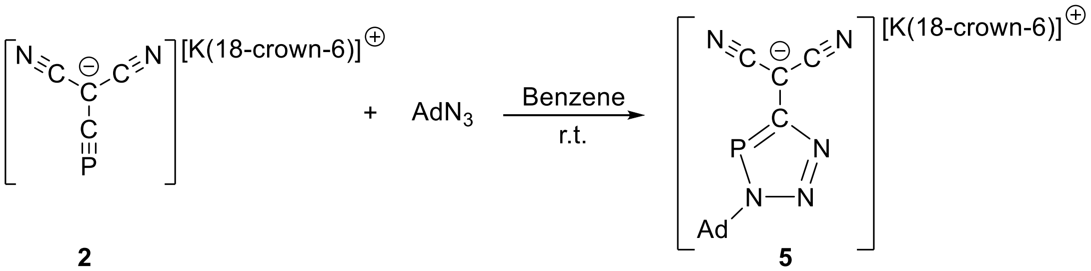

Figure S5. Synthesis of [K(18-crown-6)]**5.**

A benzene solution (0.3 mL) of AdN_3 (4.4 mg, 0.024 mmol, 1.00 eq.) was slowly added to a benzene solution (0.3 mL) of [K(18-crown-6)]**2** (10 mg, 0.024 mmol, 1.00 eq.). The mixture was stirred for 24 hours before all volatiles were removed *in vacuo* to obtain a dark brown oil. The oil was re-dissolved in THF and filtered to remove insoluble impurities. The THF solution was layered with pentane and placed in a −35 °C freezer. A brown powder was collected after 3 days and washed with pentane (2 × 1 mL), then dried *in vacuo* to afford [K(18-crown-6)]**5** (7 mg, 0.012 mmol; yield: 49%). Single crystal of [K(18-crown-6)]**5** suitable for X-ray diffraction were obtained by slow diffusion hexane into a concentrated benzene and THF solution of the product at room temperature.

Anal. Calcd. for $C_{26}H_{39}KN_5O_6P$ (M.W. 587.65 g mol^{-1}): C, 53.13; H, 6.69; N, 11.92; Found: C 53.56; H, 6.67; N, 11.30.

^{1}H NMR (600 MHz, C_6D_6): δ (ppm) 1.50 (m, 6H; C*H*$_2$ Ad), 1.91 (s, 3H; C*H* Ad), 2.26 (d, $^{4}J_{P-H}$ = 2.3 Hz, 6H; C*H*$_2$ Ad), 3.24 (s, 24H; 18-crown-6).

^{13}C{^{1}H} NMR (151 MHz, C_6D_6): δ (ppm) 29.05 (d, $^{2}J_{P-C}$ = 38.3 Hz; *C*(CN)$_2$), 30.30 (s; *C*H Ad), 36.47 (s; *C*H$_2$ Ad), 45.40 (d, $^{3}J_{P-C}$ = 6.8 Hz; *C*H$_2$ Ad), 60.80 (d, $^{2}J_{P-C}$ = 7.9 Hz; *C* Ad), 70.19 (s, 18-crown-6), 126.58 (d, $^{3}J_{P-C}$ = 3.4 Hz; *C*N), 186.88 (d, $^{1}J_{P-C}$ = 50.8 Hz; *C*P)

^{31}P NMR (162 MHz, C_6D_6): δ (ppm) 136.5 (s).

2. IR and NMR spectra

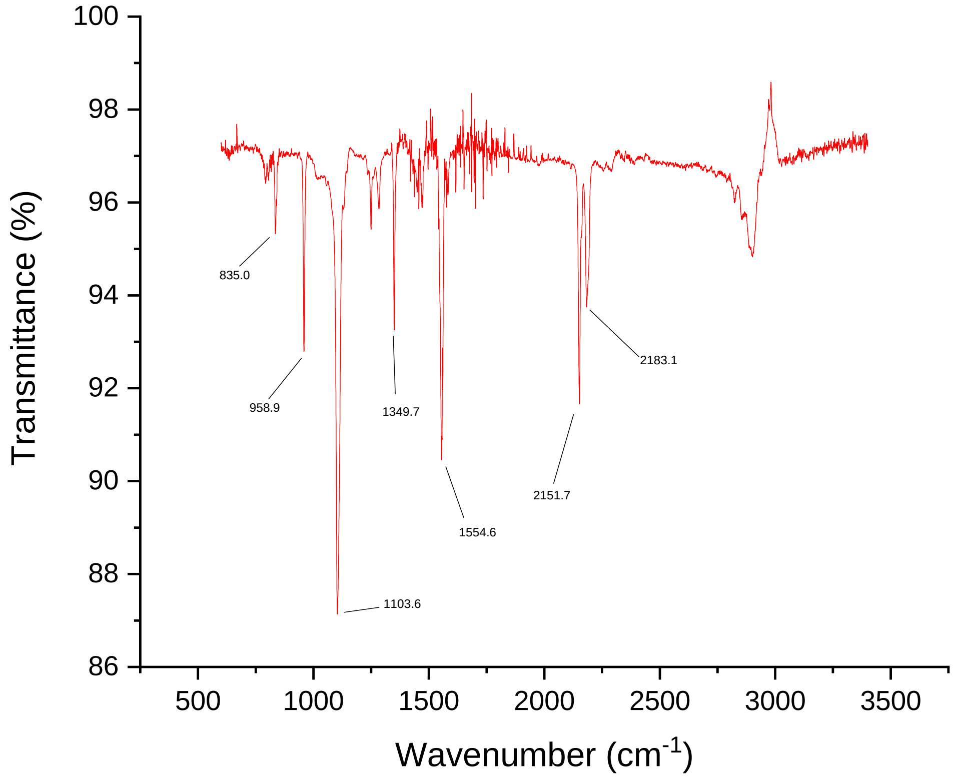

Figure S6. FTIR spectrum of [K(18-crown-6)]**2**.

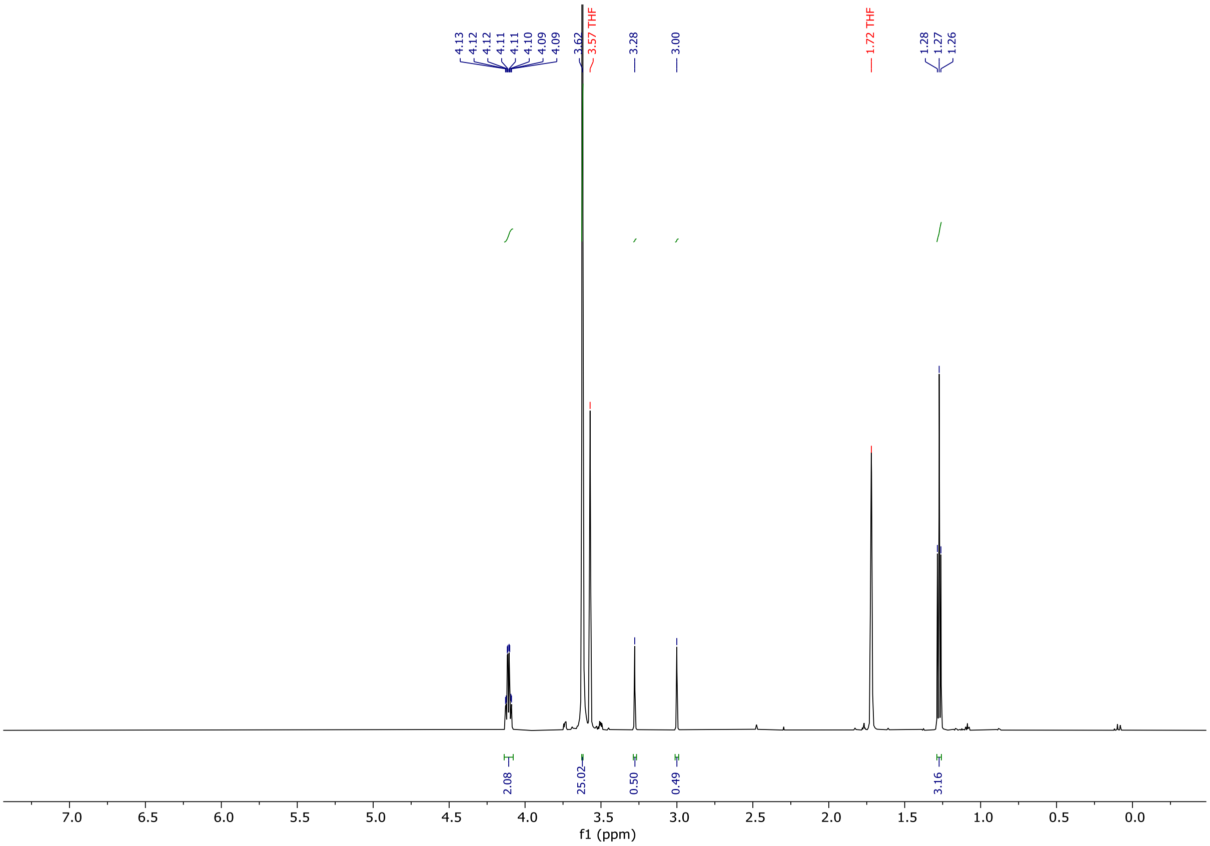

Figure S7. ^{1}H NMR spectrum of [Na(18-crown-6)]**1** (600 MHz, d_8-THF).

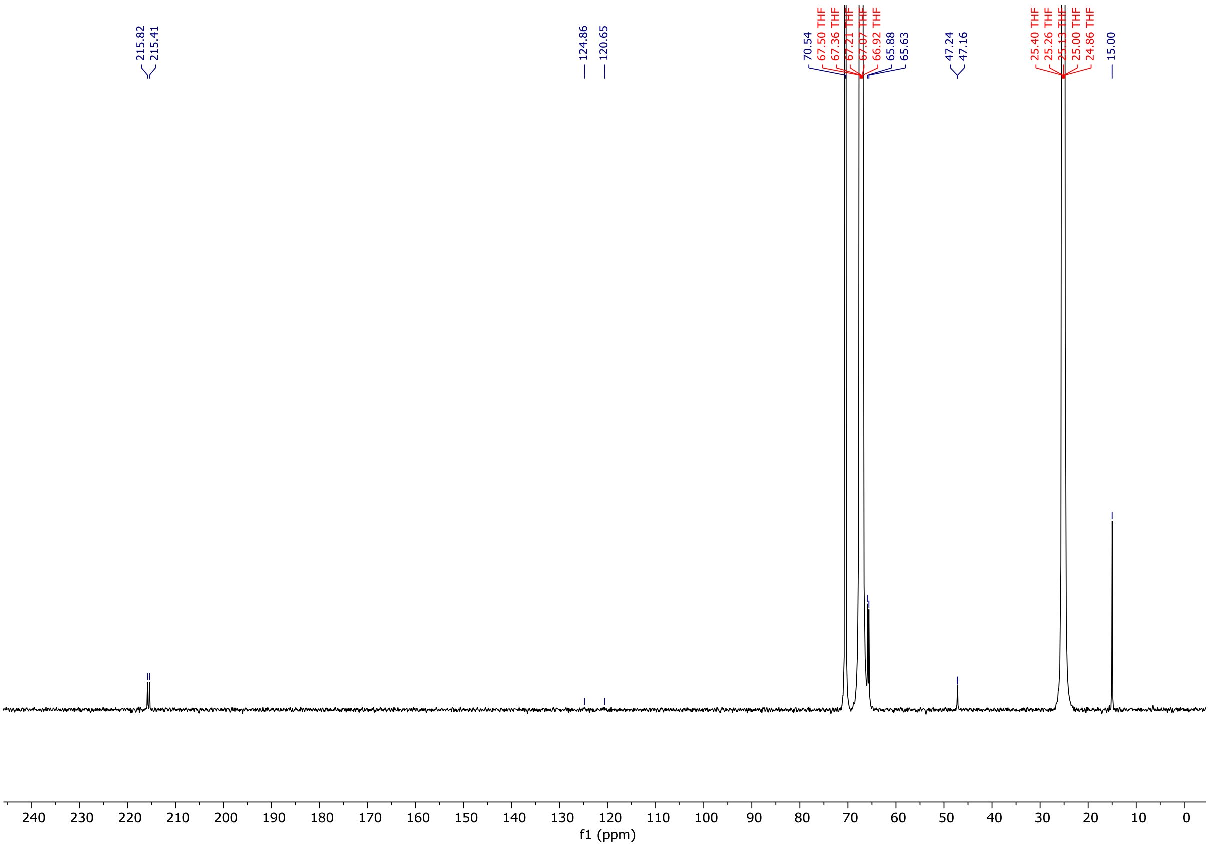

Figure S8. ^{13}C NMR spectrum of [Na(18-crown-6)]**1** (151 MHz, d_8-THF).

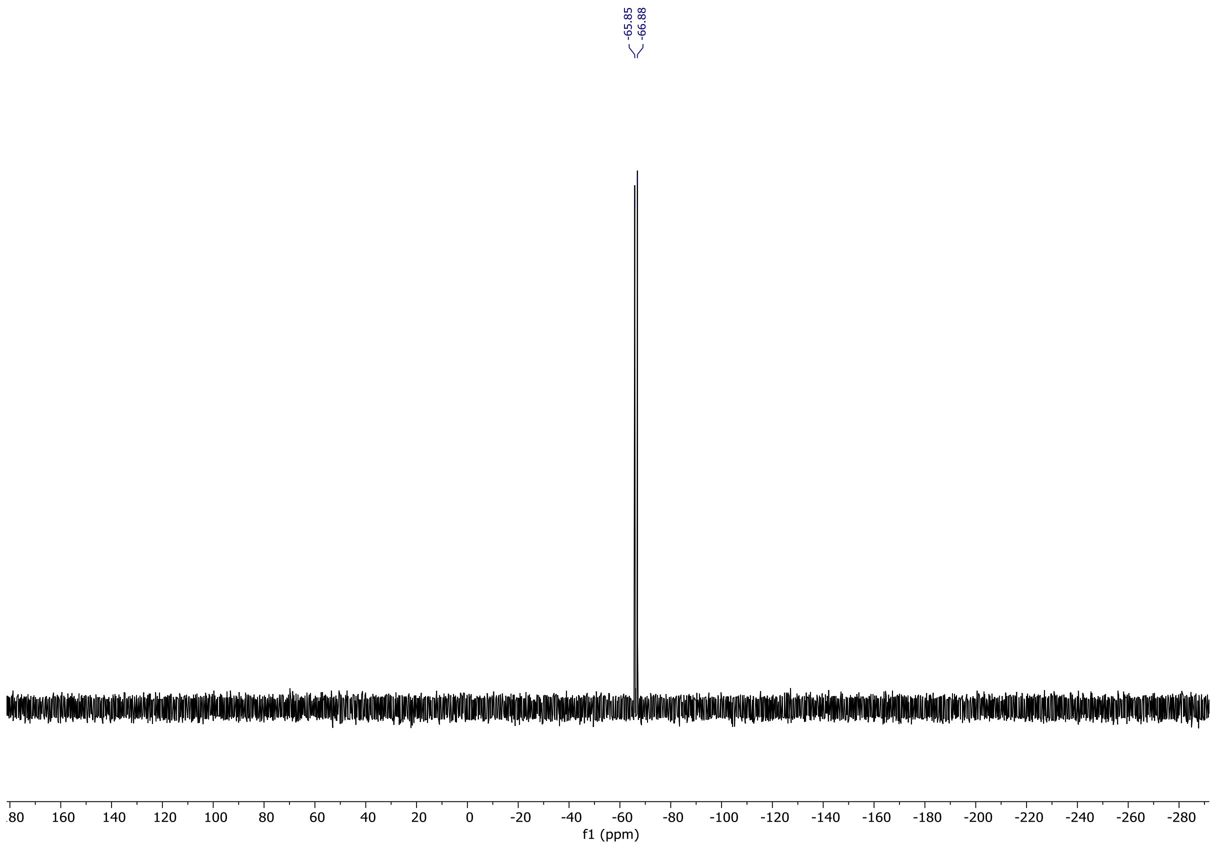

Figure S9. ^{31}P NMR spectrum of [Na(18-crown-6)]**1** (162 MHz, d_8-THF).

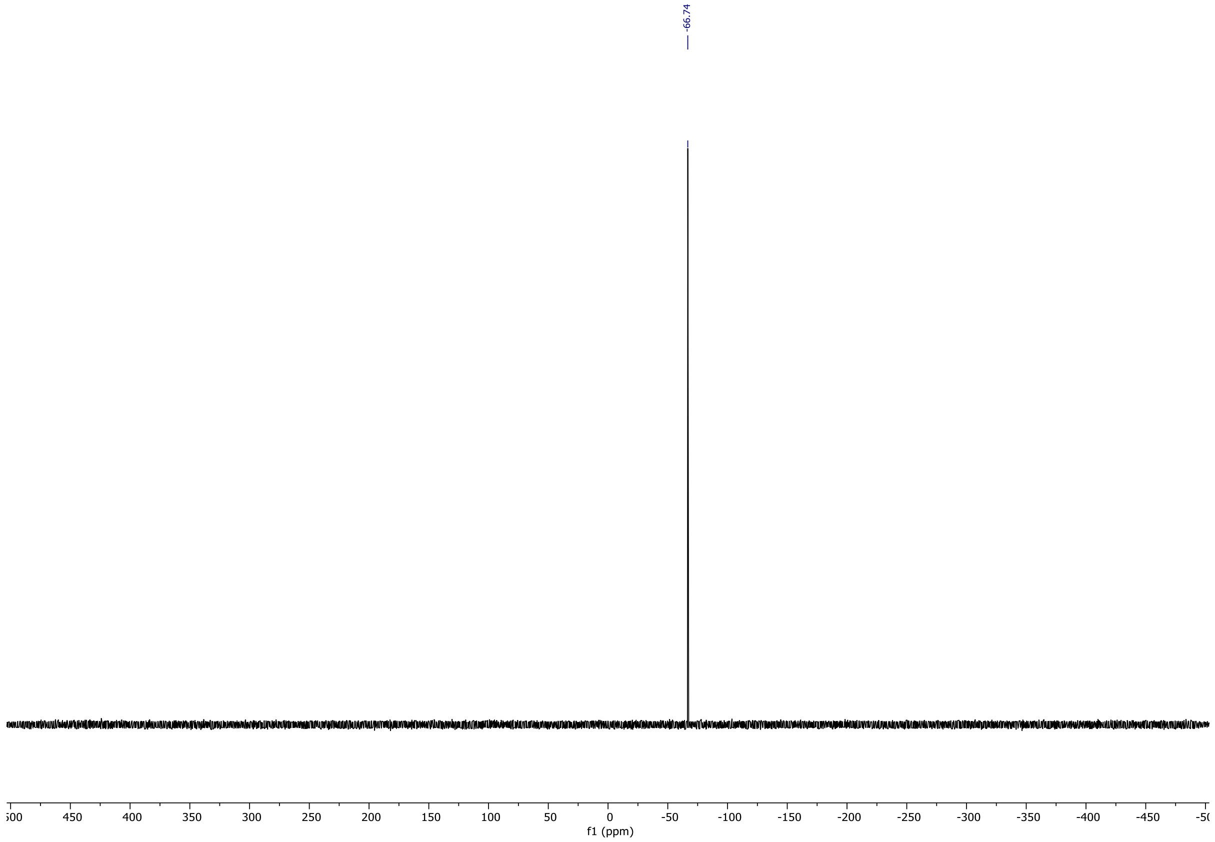

Figure S10. $^{31}P\{^1H\}$ NMR spectrum of [Na(18-crown-6)]**1** (162 MHz, d_8-THF).

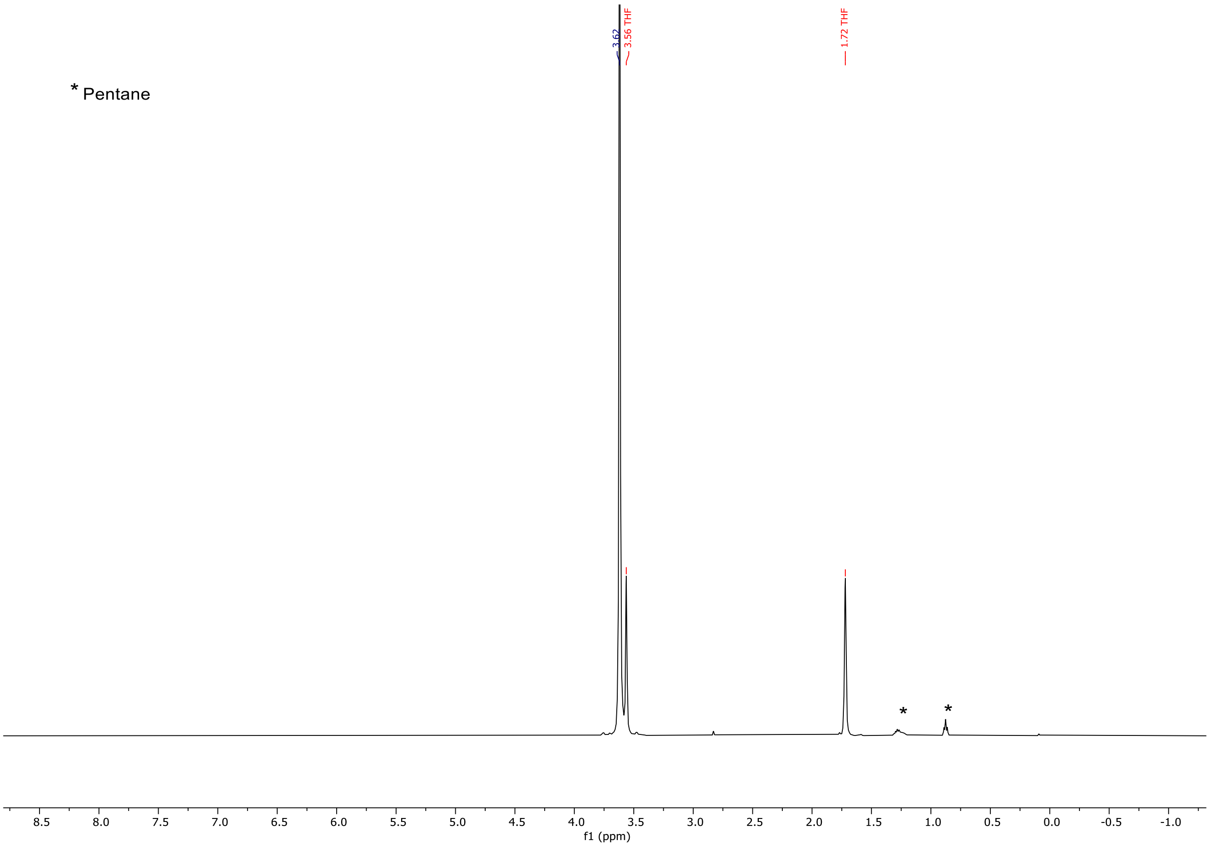

Figure S11. ^{1}H NMR spectrum of [K(18-crown-6)]**2** (500 MHz, d_8-THF).

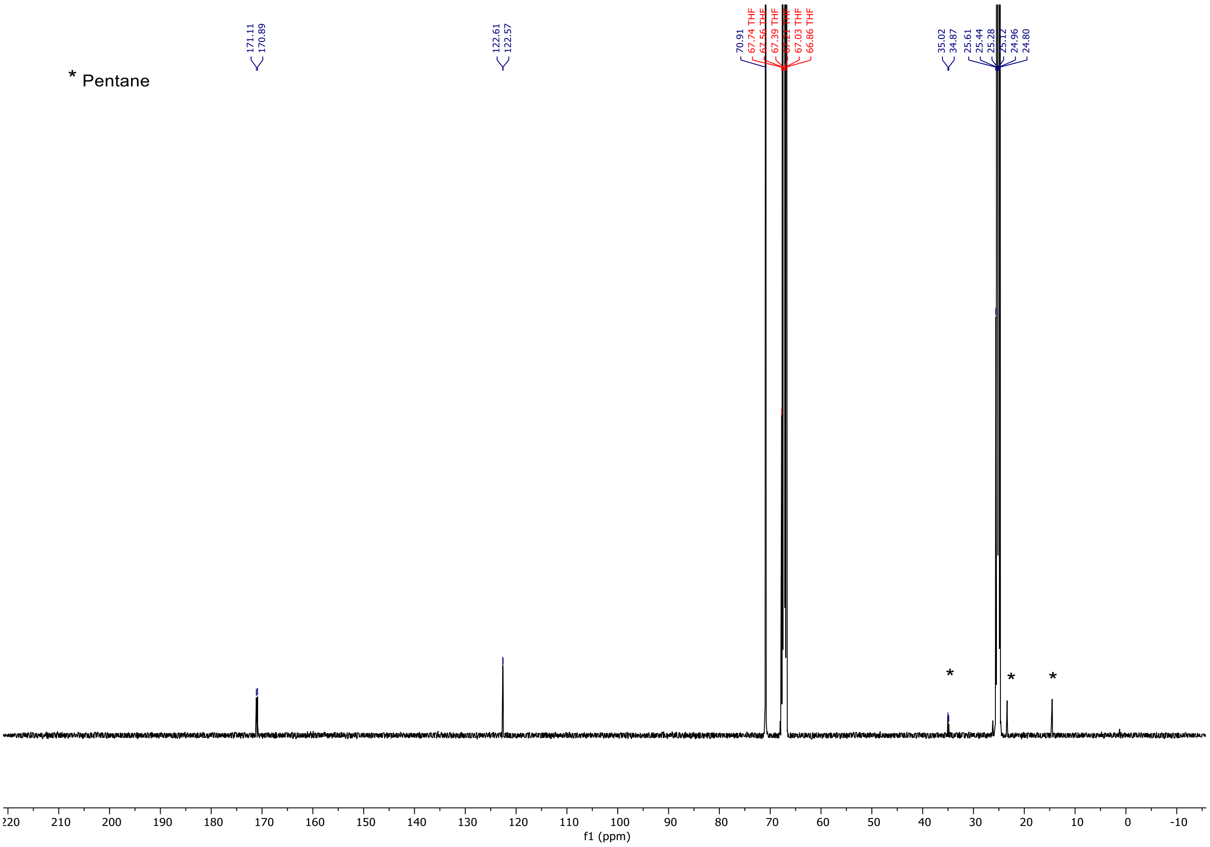

Figure S12. ^{13}C NMR spectrum of [K(18-crown-6)]**2** (125 MHz, d_8-THF).

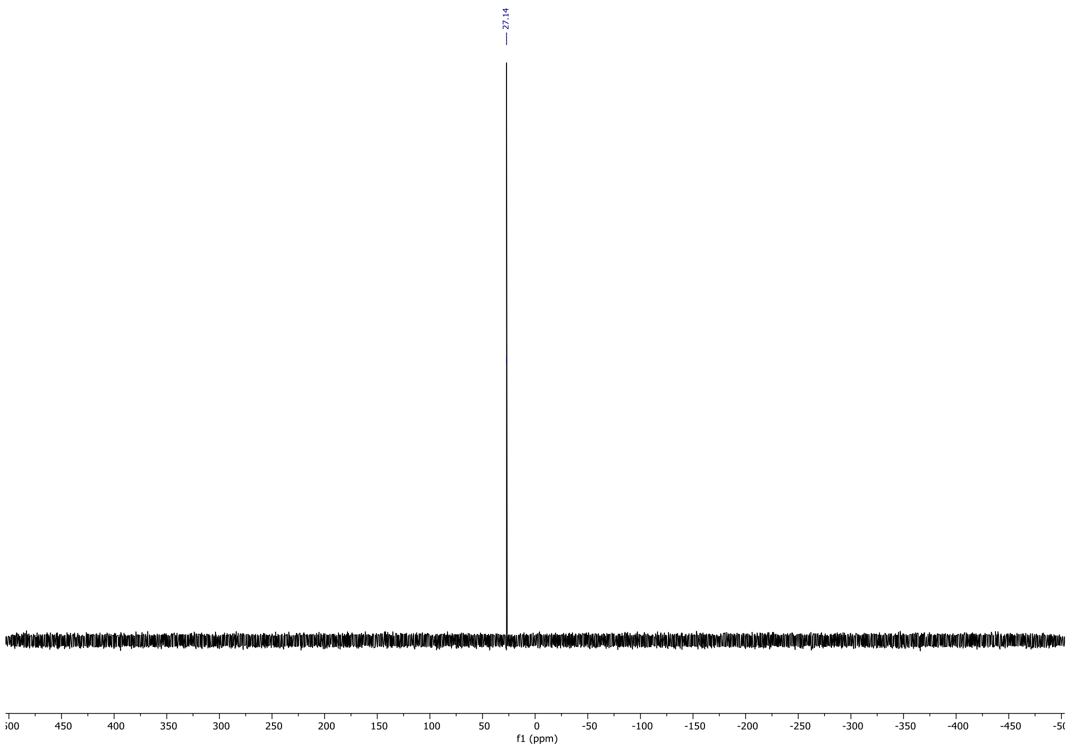

Figure S13. ^{31}P NMR spectrum of [K(18-crown-6)]**2** (162 MHz, d_8-THF).

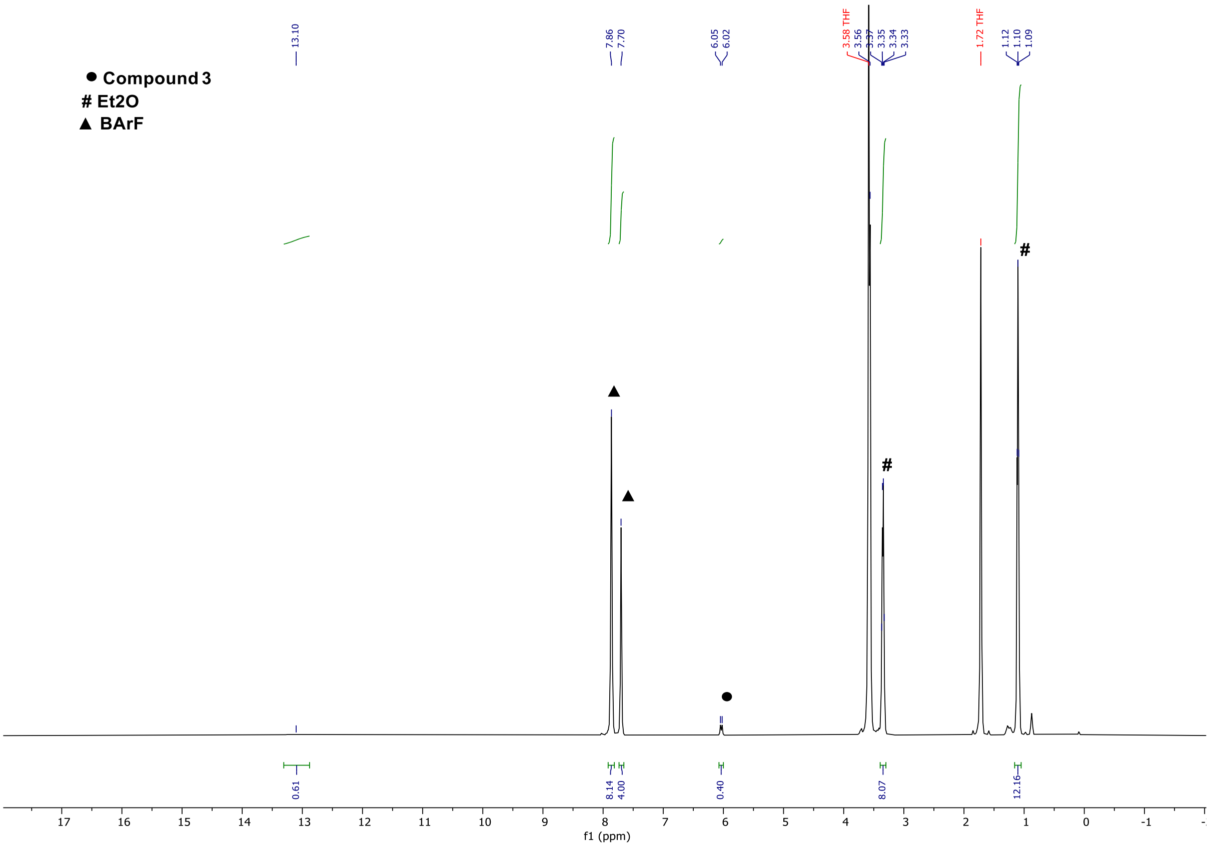

Figure S14. ^{1}H NMR spectrum of **3** (500 MHz, d_8-THF).

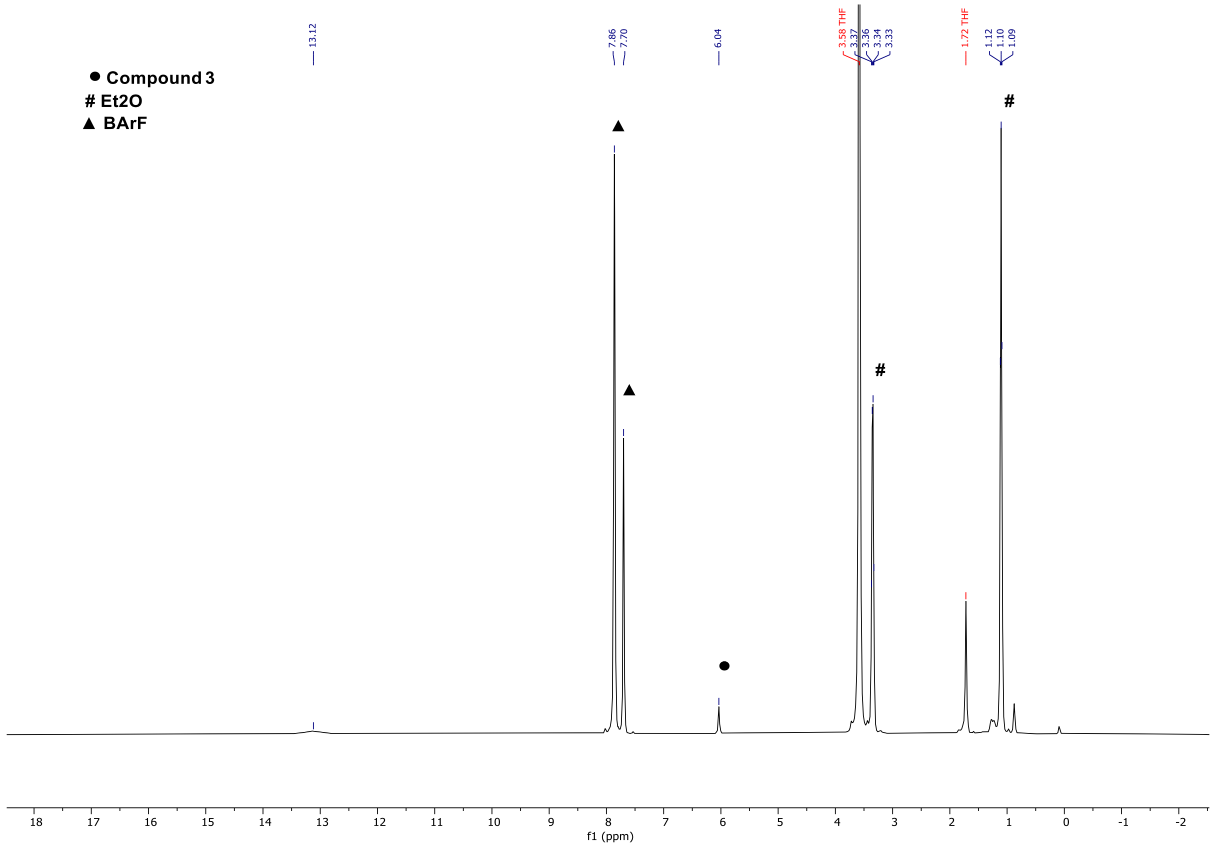

Figure S15. $^{1}H\{^{31}P\}$ NMR spectrum of **3** (500 MHz, d8-THF).

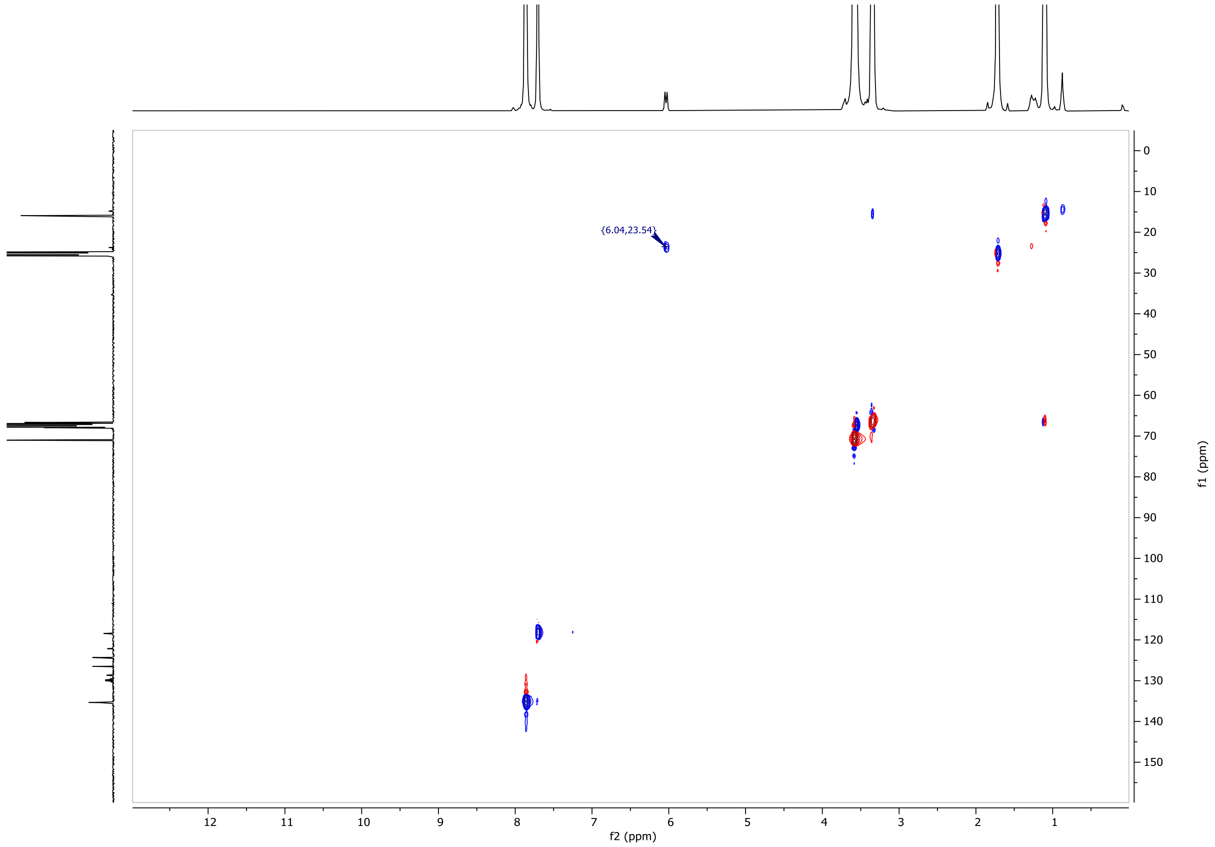

Figure S16. ^{1}H-^{13}C HMBC NMR spectrum of **3** (d8-THF).

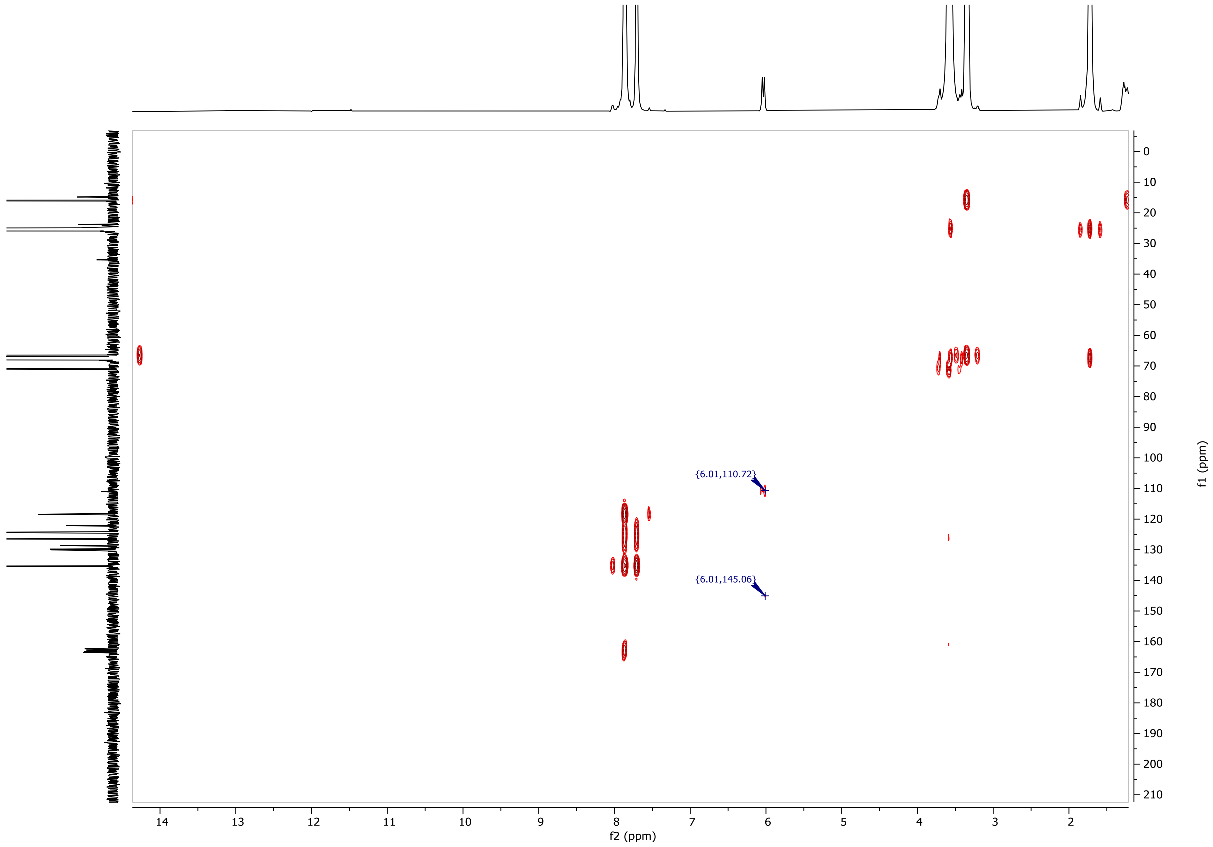

Figure S17. ^{1}H-^{13}C HSQC NMR spectrum of **3** (d_8-THF).

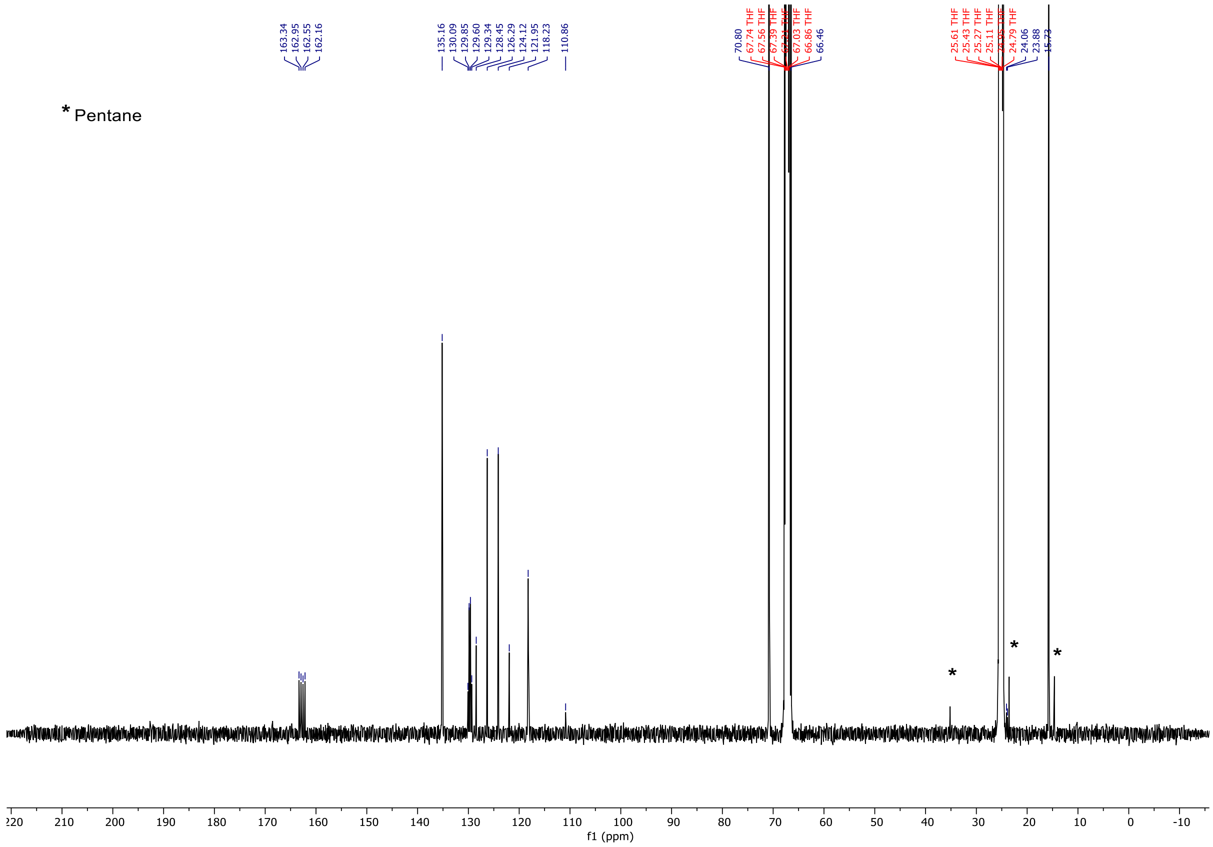

Figure S18. ^{13}C NMR spectrum of **3** (125 MHz, d_8-THF).

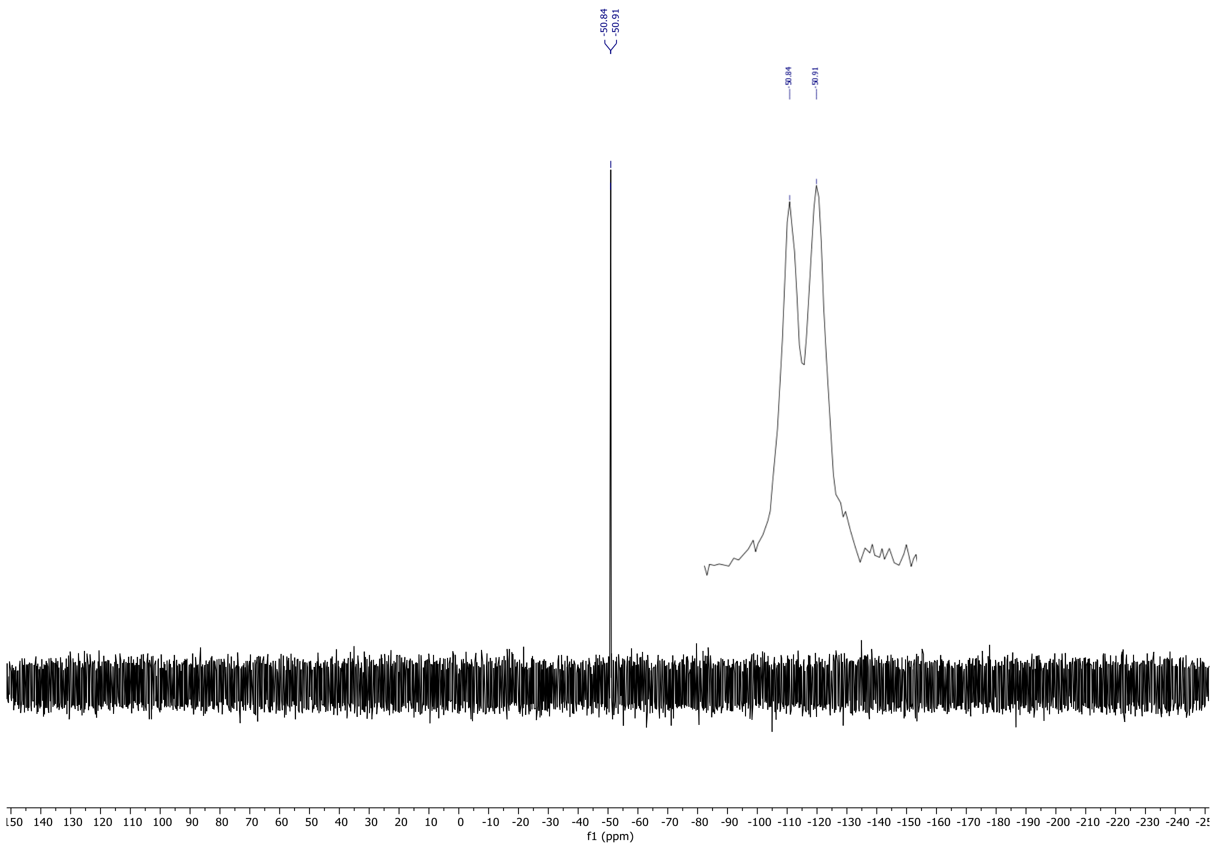

Figure S19. ^{31}P NMR spectrum of **3** (202 MHz, d_8-THF).

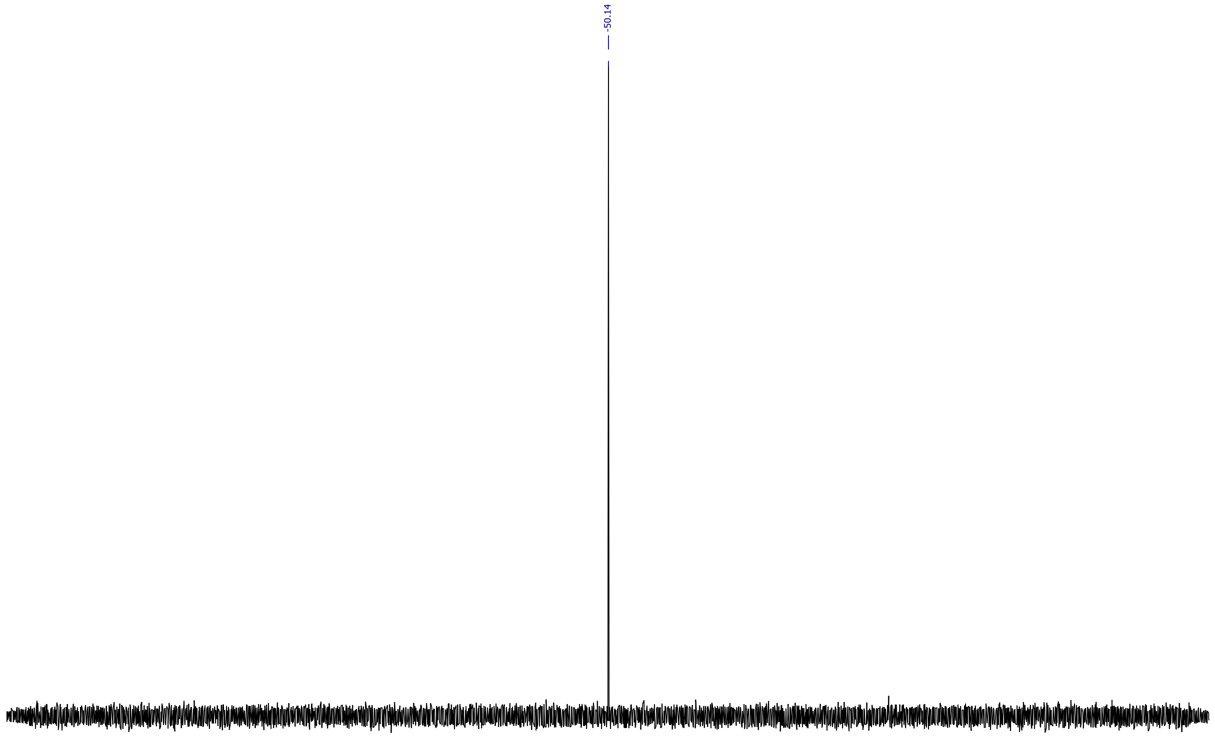

Figure S20. $^{31}P\{^{1}H\}$ NMR spectrum of **3** (202 MHz, d_8-THF).

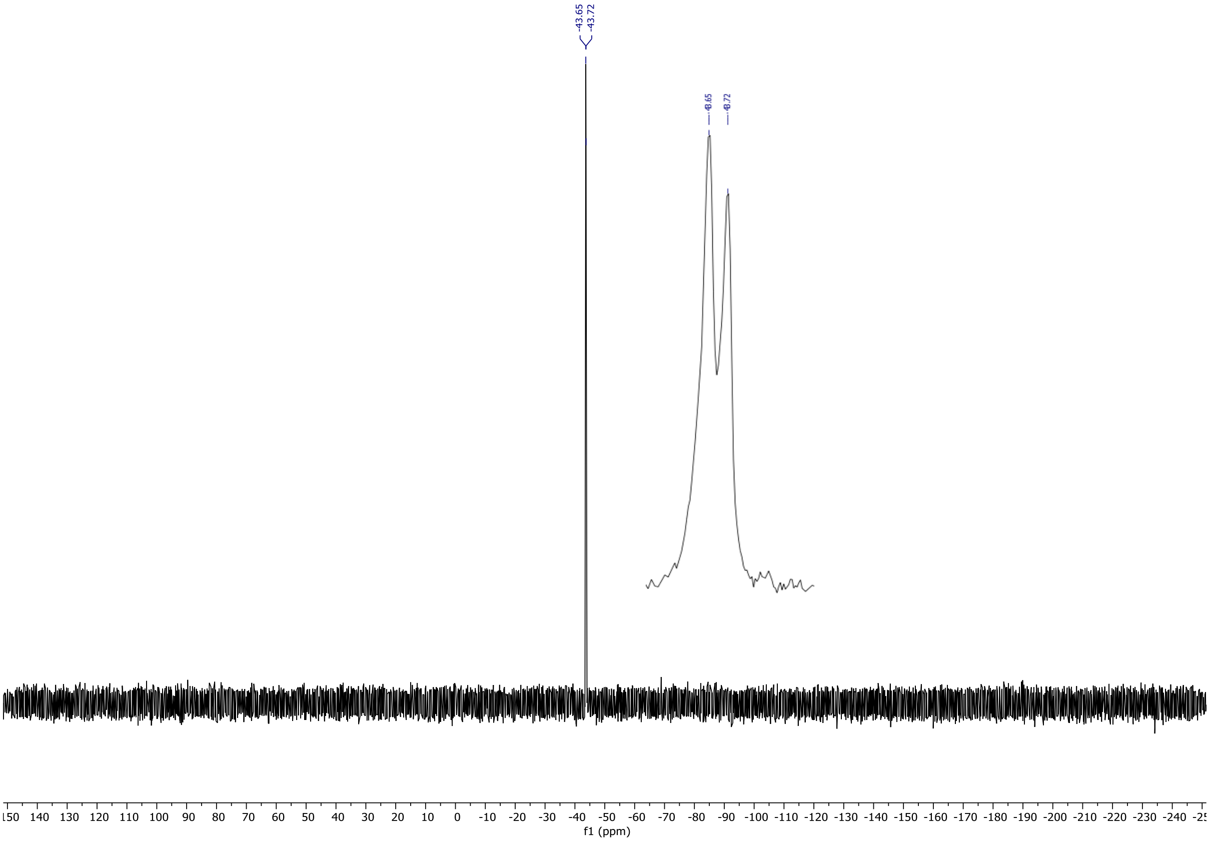

Figure S21. ^{31}P NMR spectrum of **3** (202 MHz, Et_2O).

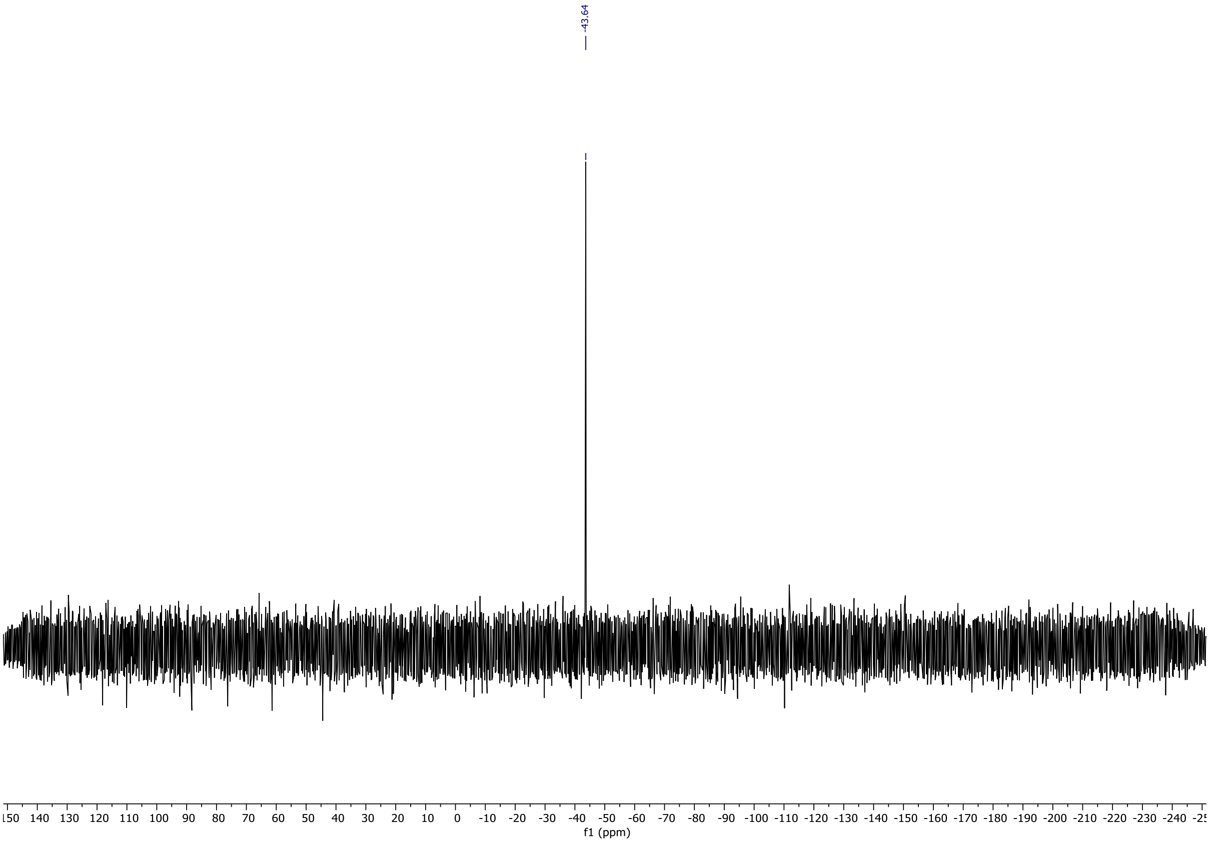

Figure S22. $^{31}P\{^1H\}$ NMR spectrum of **3** (202 MHz, Et_2O).

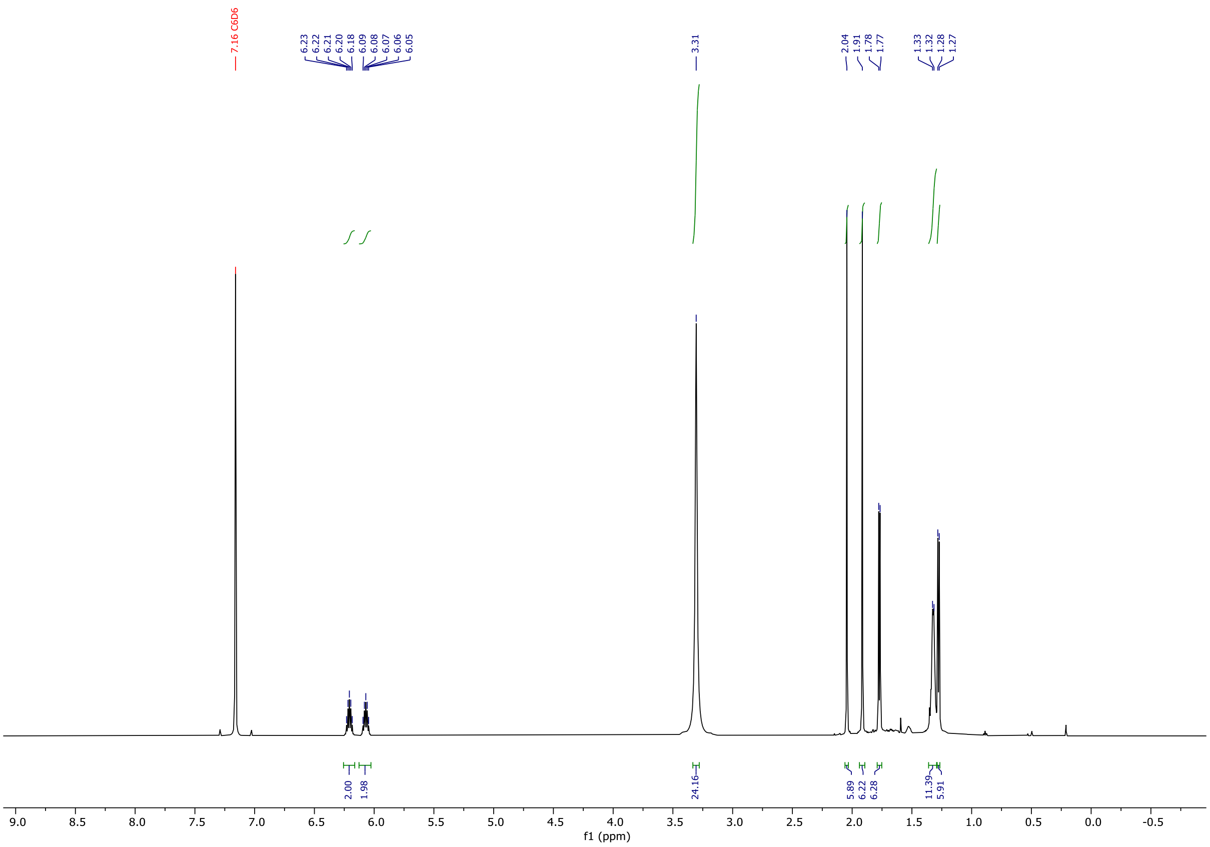

Figure S23. ^{1}H NMR spectrum of [K(18-crown-6)]**4** (600 MHz, C_6D_6).

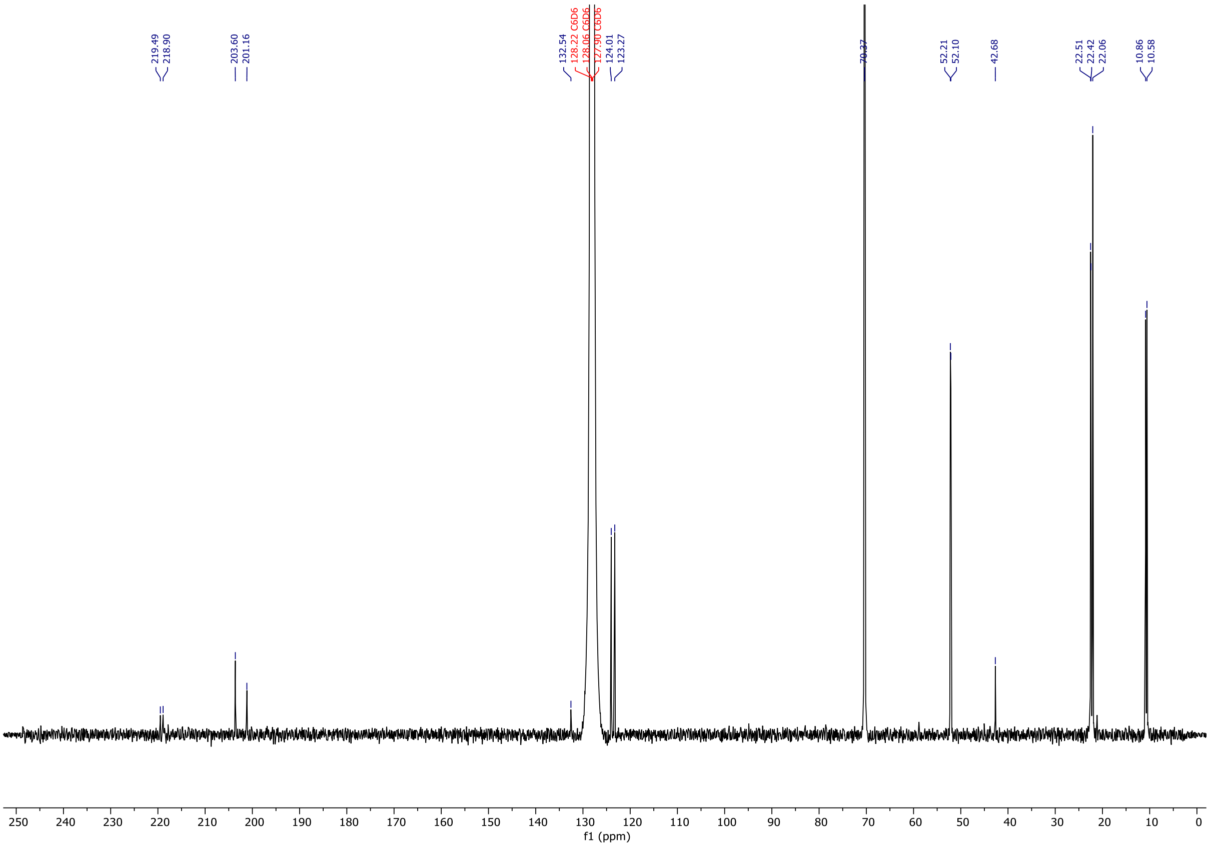

Figure S24. ^{13}C NMR spectrum of [K(18-crown-6)]**4** (151 MHz, C_6D_6).

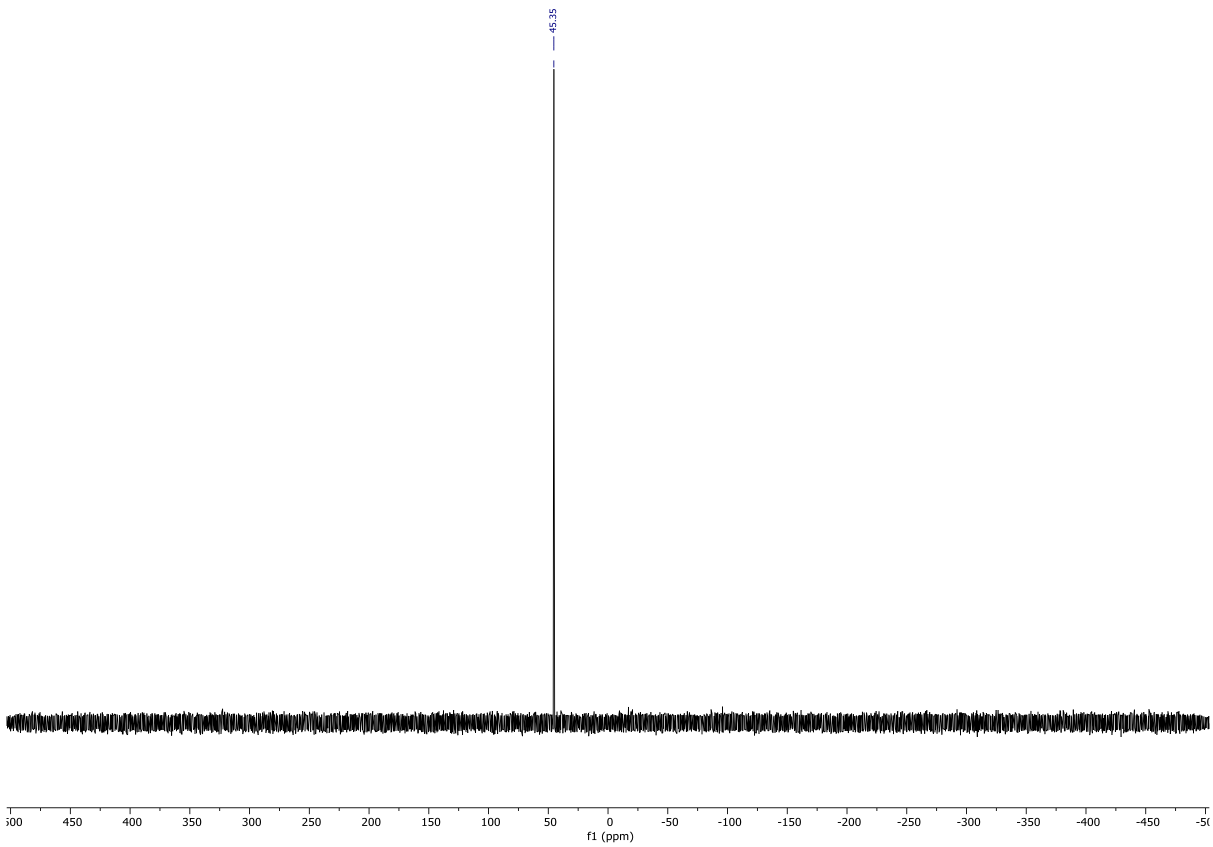

Figure S25. ^{31}P NMR spectrum of [K(18-crown-6)]**4** (162 MHz, C_6D_6).

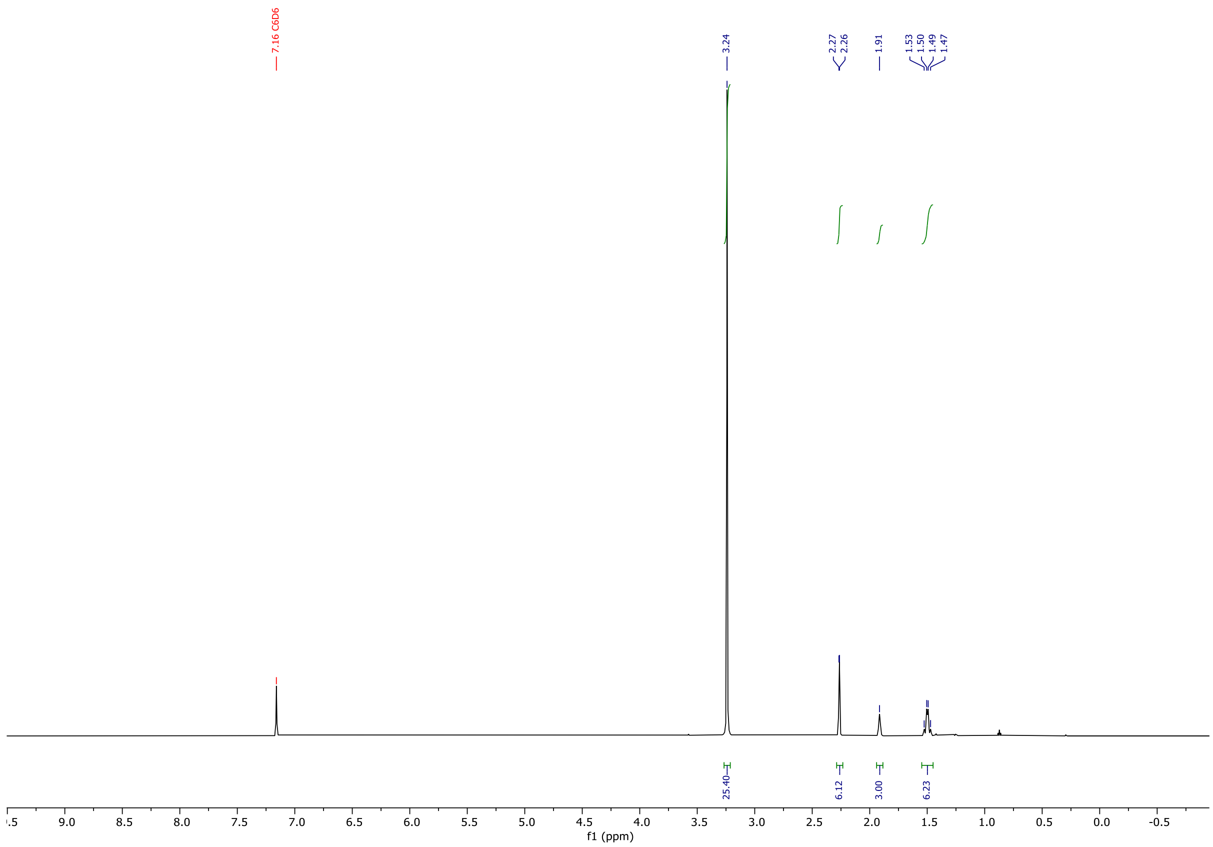

Figure S26. ^{1}H NMR spectrum of [K(18-crown-6)]**5** (600 MHz, C_6D_6).

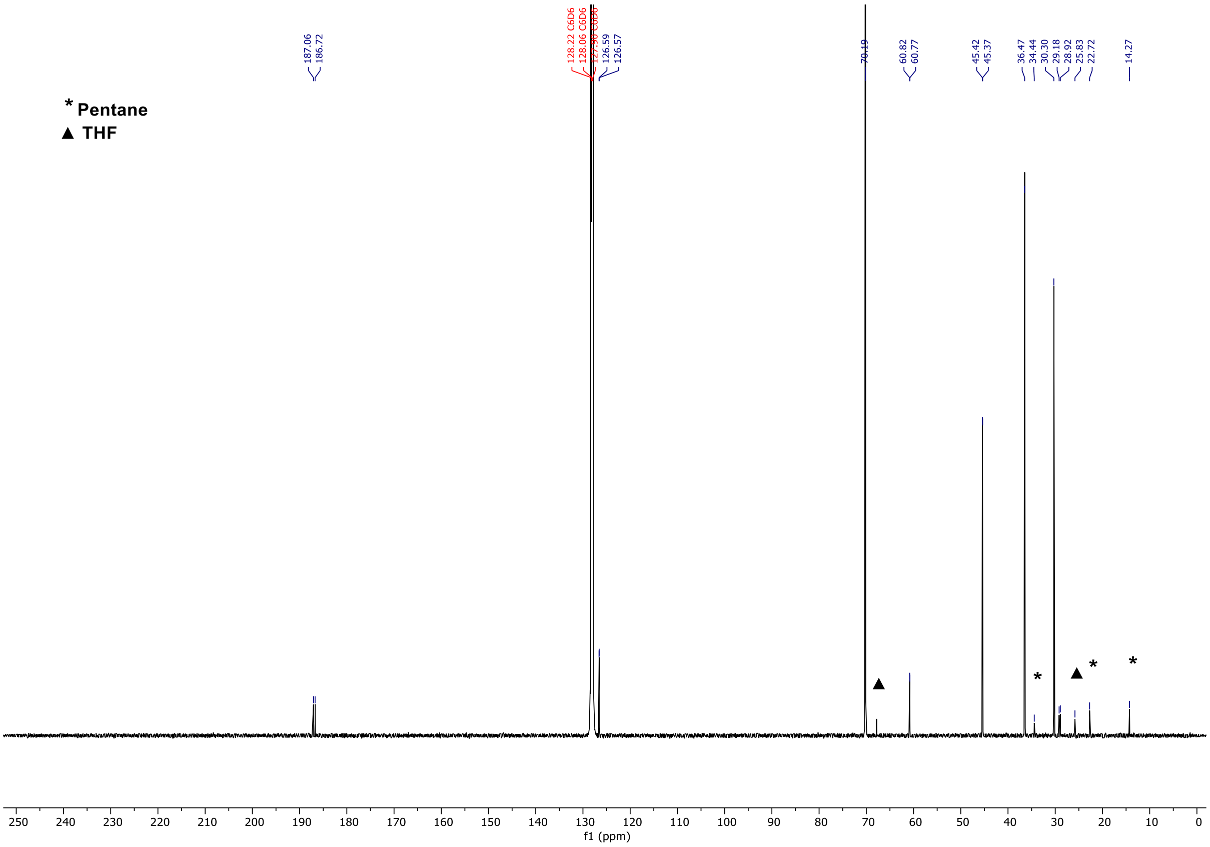

Figure S27. ^{13}C NMR spectrum of [K(18-crown-6)]**5** (151 MHz, C_6D_6).

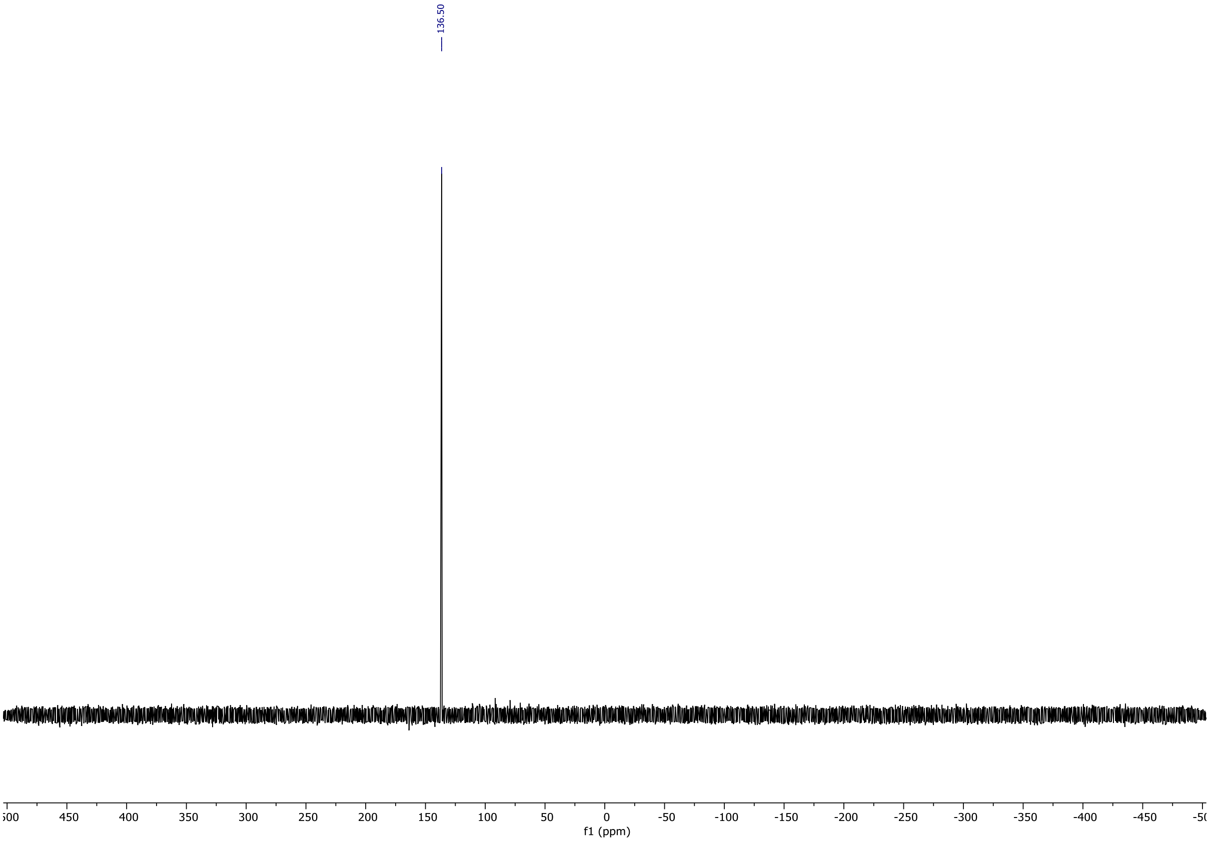

Figure S28. ^{31}P NMR spectrum of [K(18-crown-6)]**5** (162 MHz, C_6D_6).

3. X-ray Crystallographic Data

Single-crystal X-ray diffraction data were collected using an Oxford Diffraction Supernova dual-source diffractometer equipped with a 135 mm Atlas CCD area detector. Crystals were selected under Paratone-N oil, mounted on micromount loops and quench-cooled using an Oxford Cryosystems open flow N_2 cooling device. Data were collected at 150 K using mirror monochromated Cu Kα (λ = 1.54184 Å) radiation and processed using the CrysAlisPro package, including unit cell parameter refinement and inter-frame scaling (which was carried out using SCALE3 ABSPACK within CrysAlisPro).[5] Structures were subsequently solved using direct methods.[6]

Table S1. Selected X-ray data collection/refinement parameters for [Na(18-crown-6)]**1**·THF, [K(18-crown-6)]**2**·THF, [K(18-crown-6)]**4**·1.5tol and [K(18-crown-6)]**5**.

	[Na(18-crown-6)]**1**·THF	[K(18-crown-6)]**2**·THF	[K(18-crown-6)]**4**·1.5tol	[K(18-crown-6)]**5**
Formula	$C_{22}H_{38}N_2NaO_8P$	$C_{20}H_{32}KN_2O_7P$	$C_{48.5}H_{76}KN_6NiO_6P$	$C_{26}H_{39}KN_5O_6P$
CCDC	2179806	2179807	2179808	2179809
Fw [g mol^{-1}]	512.50	482.54	967.93	587.69
Crystal system	triclinic	triclinic	monoclinic	triclinic
Space group	$P\overline{1}$	$P\overline{1}$	$P2_1/n$	$P\overline{1}$
a (Å)	9.0861(3)	8.0042(5)	11.1847(1)	10.1564(3)
b (Å)	9.1743(3)	9.8316(4)	33.9741(2)	10.7107(3)
c (Å)	16.4208(4)	16.1020(8)	13.9174(1)	15.3083(4)
α (°)	87.976(2)	95.380(4)	90	75.303(2)
β (°)	82.419(2)	100.530(4)	91.247(1)	82.672(2)
γ (°)	89.177(2)	91.780(4)	90	65.466(3)
V (Å^3)	1355.94(7)	1238.80(11)	5287.22(7)	1464.89(8)
Z	2	2	4	2
Radiation, λ (Å)	Cu Kα, 1.54184	Cu Kα, 1.54184	Cu Kα, 1.54184	Cu Kα, 1.54184
Temp (K)	150(2)	150(2)	150(2)	150(2)
ρ_{calc} (g cm^{-3})	1.255	1.294	1.216	1.332
μ (mm^{-1})	1.446	2.837	1.915	2.503
Reflections collected	31042	21813	92959	31548
Independent reflections	5617	5658	10987	6082
Parameters	312	353	650	439
R(int)	0.0389	0.0424	0.0458	0.0315
R1/wR2,[a] I ≥ 2σI (%)	4.34/12.27	4.39/10.04	3.16/8.54	3.00/7.47
R1/wR2,[a] all data (%)	5.23/13.08	6.69/11.21	3.66/8.93	3.50/7.86
GOF	1.064	1.040	1.031	1.037

[a] $R1 = [\Sigma||F_o| - |F_c||]/\Sigma|F_o|$; $wR2 = \{[\Sigma w[(F_o)^2 - (F_c)^2]^2]/[\Sigma w(F_o^2)^2\}^{1/2}$; $w = [\sigma^2(F_o)^2 + (AP)^2 + BP]^{-1}$, where $P = [(F_o)^2 + 2(F_c)^2]/3$ and the A and B values are 0.0734 and 0.31 for [Na(18-crown-6)]**1**·THF, [K(18-crown-6)]**2**·THF, 0.0501 and 0.99 for [K(18-crown-6)]**4**·1.5tol, and 0.0367 and 0.44 for [K(18-crown-6)]**5**.

4. Computational Details

Geometry optimizations were carried out using the Gaussian 16 package with the M06-2X functional.[7,8] The def2-SVP basis set was used for all the atoms.[9,10] Frequency calculations at the same level of theory were performed to identify the number of imaginary frequencies (zero for local minimum and one for transition states), and provide the thermal corrections of Gibbs free energy. Single-point energy calculations were performed at the M06-2X/def2-TZVP level of theory for the modelling of compounds in solution (THF). The gas-phase geometry was used for all the solution phase calculations. The SMD method was used with the corresponding solvent, while Bondi radii were chosen as the atomic radii to define the molecular cavity.[11] The corrections of Gibbs free energy from frequency calculations were added to the single-point energies to obtain the Gibbs free energy in solution. All the energies reported in the paper correspond to the reference state of 1 mol/L, 298K. Natural bond orbital (NBO) calculations were carried out using NBO 7.0 program at the M06-2X/def2-TZVP level of theory.[12] Natural Resonance Theory was used as implemented in NBO 7.0. Isotropic shifts for **3** were computed at the GIAO-PBE1PBE/6-311G(d,p)][13–17] level of theory using the phosphorus (−69.2 ppm) and carbon atoms (184.8ppm) of ^{t}BuCP as reference.[18] No solvent corrections were applied. Optimized structures were visualized using Chemcraft.[19]

4.1. Molecular Orbitals (MO)

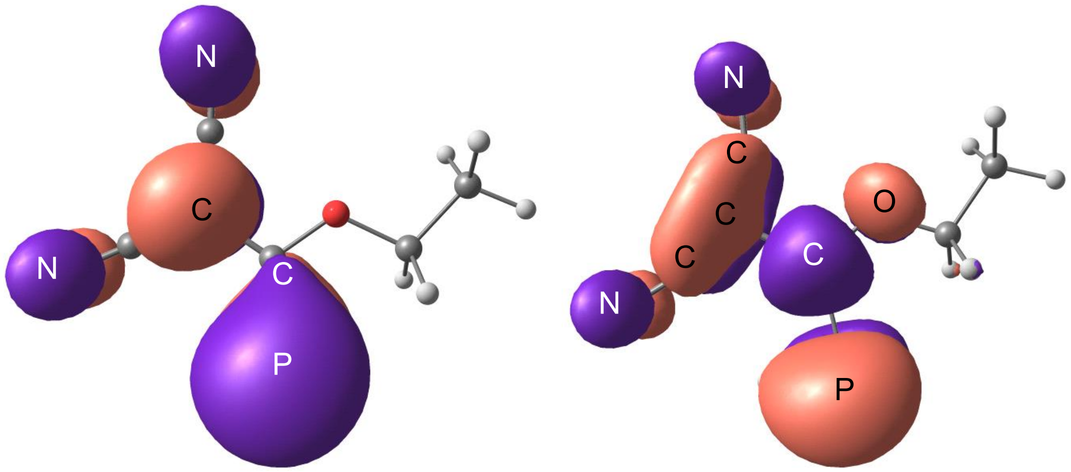

HOMO (−2.01 eV) **LUMO (4.41 eV)**

Figure S29. Kohn-Sham orbital depictions of the HOMO and LUMO of $[HP\{C(OEt){=}C(CN)_2\}]^-$ rendered at an isovalue of 0.03.

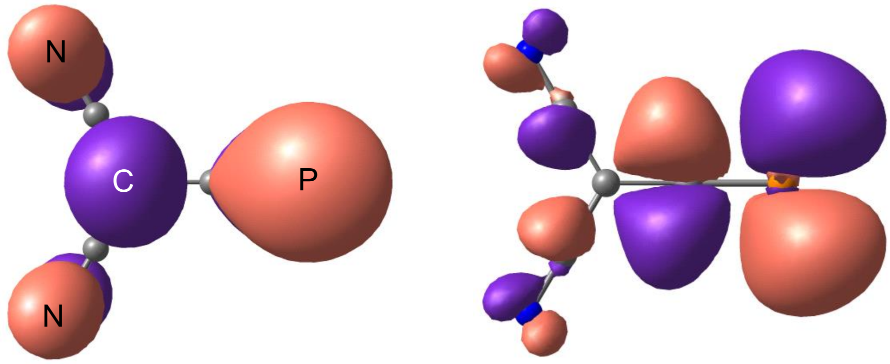

HOMO (−2.19 eV) **LUMO (3.86 eV)**

Figure S30. Kohn-Sham orbital depictions of the HOMO and LUMO of $[C(CN)_2(CP)]^-$ rendered at an isovalue of 0.03.

4.2. Natural bond orbital (NBO) analysis of compound 1 and 2

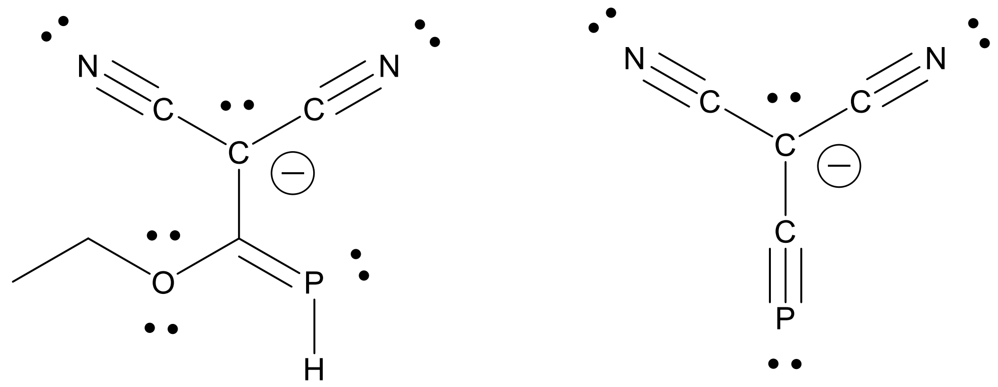

Figure S31. NBO assigned Lewis structures of compounds [HP{C(OEt)=C(CN)$_2$}]$^-$ and [C(CN)$_2$(CP)]$^-$.

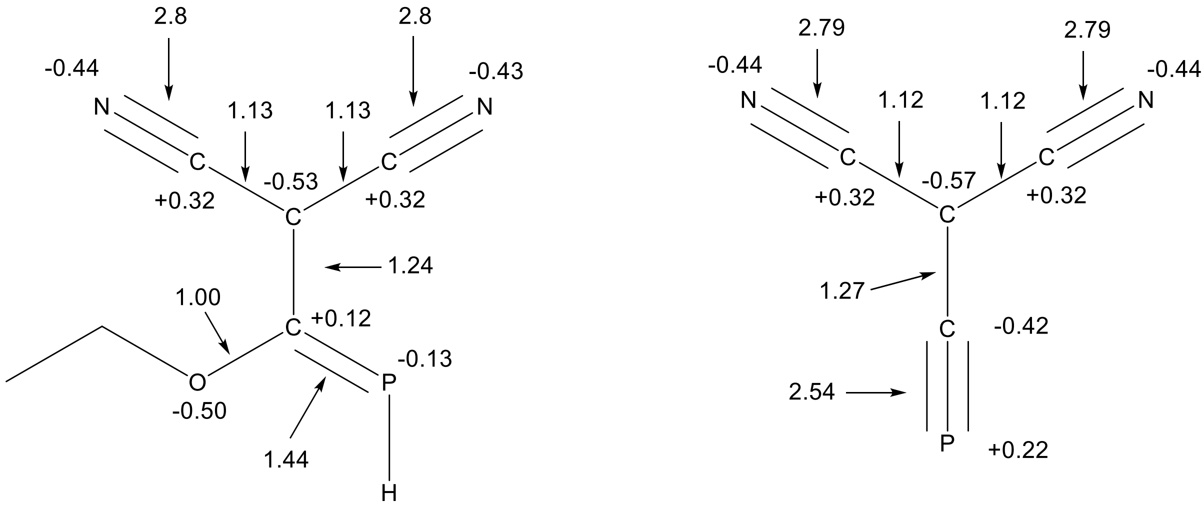

Figure S32. Wiberg Bond Order and NPA charges of [HP{C(OEt)=C(CN)$_2$}]$^-$ and [C(CN)$_2$(CP)]$^-$.

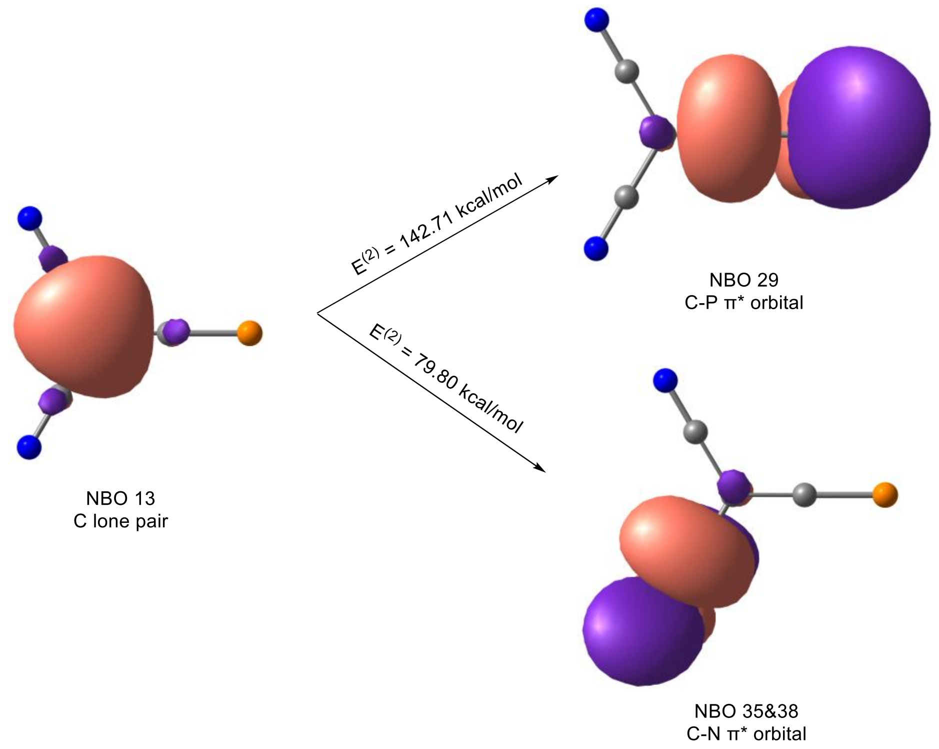

Figure S33. Selected NBOs of $[C(CN)_2(CP)]^-$ from second-order perturbation theory analysis.

4.3. Relative energy of isomers of 3

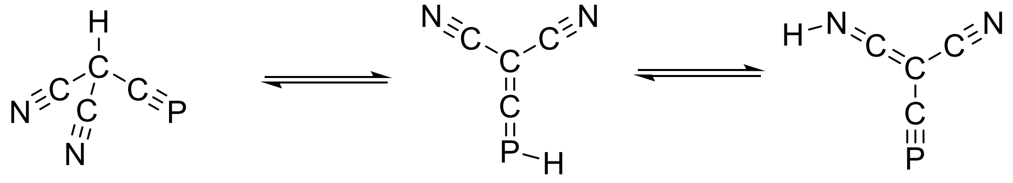

0.0 kcal/mol 5.0 kcal/mol 6.9 kcal/mol

3 **3-PH** **3-NH**

Figure S34. Relative energy of **3** and its isomers.

Table S5. Energies of **3** and its isomers.

Species	Thermal Corrections of Gibbs Free Energies (Hartree)	Solvation Energies (Hartree)
**3**	0.009379	−603.774544
**3-PH**	0.006011	−603.763171
**3-NH**	0.008753	−603.763733

4.4. Comparison Between $[C(CP)(CN)_2]^-$ and $[C(CN)_3]^-$

4.4.1 Natural Resonance theory (NRT) Analysis

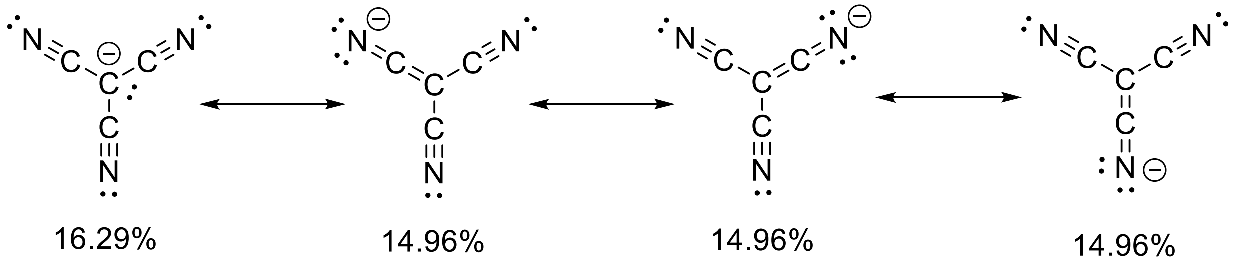

Figure S35. The four major resonance structures of $[C(CN)_3]^-$ as predicted by natural resonance theory (NRT).

Unlike $[C(CP)(CN)_2]^-$, the resonance structure of $[C(CN)_3]^-$ with a lone pair on the central carbon atom (and a formal negative charge) has the greatest contribution to the overall electronic structure. This is consistent with our findings that the cyaphide moiety can delocalize negative charge better than a nitrile group. Here, without the cyaphide moiety, more negative charge accumulates on the central carbon, causing this resonance structure to become the most significant one. But since the cyaphide moiety is only slightly better at delocalizing negative charge, the weight of each of the other resonance structures is similar to those calculated for $[C(CP)(CN)_2]^-$.

4.4.2 NPA charge comparison

Table S6. NPA charge of $[C(CP)(CN)_2]^-$ and $[C(CN)_3]^-$.

**Atom type**	$\mathbf{[C(CP)(CN)_2]^-}$	$\mathbf{[C(CN)_3]^-}$
**Central C**	−0.57	−0.61
**N**	−0.44	−0.44
**CN Moiety**	−0.12	−0.13
**P**	+0.22	
**CP Moiety**	−0.20	

The NPA charges show that the central carbon in $[C(CN)_3]^-$ carries more negative charge than in $[C(CP)(CN)_2]^-$. This finding, again, agrees with our hypothesis that cyaphide moiety can delocalize negative charge better than a nitrile group.

4.4.3 Wiberg bond order comparison

Table S7. Wiberg bond order of $[C(CP)(CN)_2]^-$ and $[C(CN)_3]^-$.

**Bond Type**	$\mathbf{[C(CP)(CN)_2]^-}$	$\mathbf{C(CN)_3^-}$
**C–CN**	1.12	1.15
**CN**	2.79	2.77
**C–CP**	1.27	
**CP**	2.54	

4.5. Cartesian Coordinates

1:

C	-0.01037900	-0.45357600	-0.08098900
C	0.54345900	0.85882500	-0.11829100
C	-1.40753900	-0.68684600	-0.14658700
C	0.83900200	-1.58903600	0.02437900
N	-2.55046500	-0.89266300	-0.19944700

N	1.51839900	-2.52825100	0.11050100
O	1.89726300	0.81479600	-0.04392000
C	2.62946000	2.01299300	-0.06332200
H	2.31260000	2.65755200	0.77614700
H	2.40984300	2.56963600	-0.99202700
C	4.09684800	1.65295200	0.03522500
H	4.38759400	1.01143700	-0.80789700
H	4.71845100	2.55951400	0.02446900
H	4.28998900	1.09940800	0.96431700
P	-0.26912000	2.40669400	-0.24080000
H	-1.56832800	1.83211100	-0.27723600

2:

C	-2.18198400	0.27212600	0.76257100
P	-1.19186400	0.92794800	1.78567000
C	-3.06477500	-0.31447000	-0.14655600
C	-3.25787400	0.24723400	-1.44001500
C	-3.79230400	-1.48751900	0.20054600
N	-4.38367600	-2.44558900	0.49016500
N	-3.41148300	0.71069100	-2.49505600

3:

C	-2.22708900	0.40340700	0.81175500
P	-1.00595000	0.84449800	1.62894300
C	-3.40638500	-0.02238000	0.02579600
C	-3.23848300	0.28415900	-1.41054300
C	-3.69675600	-1.45843800	0.22239100
N	-3.92980000	-2.57191400	0.39766600
N	-3.11018600	0.54568800	-2.52409500
H	-4.29080200	0.53902300	0.37670100

3-PH:

C	-2.18252400	0.32217000	0.73687300
P	-1.28718900	1.12266300	1.83886800
C	-3.01166200	-0.26595400	-0.12136400
C	-3.23074600	0.28902100	-1.43633000
C	-3.70240200	-1.48328100	0.23366500
N	-4.25036400	-2.45619700	0.52373500
N	-3.40094600	0.73690100	-2.48580700
H	-0.07724900	0.45692600	1.47220500

3-NH:

C	-2.20850400	0.22722300	0.80823900
P	-1.28478700	0.91098400	1.83781200
C	-3.06288300	-0.39444300	-0.15562100
C	-3.31218000	0.20929000	-1.43696500
C	-3.67051900	-1.55864600	0.13491900
N	-4.10821500	-2.65432700	0.36262700
N	-3.51253900	0.70125600	-2.46247900
H	-5.01669900	-2.80062300	0.80808500

^{t}BuCP:

C	-2.23303100	0.40095700	0.80828300
P	-1.00510500	0.84492400	1.62892100
C	-3.40774000	-0.02303000	0.02495000
C	-4.56966400	-0.32903200	0.98621200
H	-5.45250300	-0.64660000	0.41086500
H	-4.29647400	-1.13364800	1.68292500
H	-4.83442400	0.56194000	1.57233100
C	-3.04826200	-1.28502000	-0.77865900
H	-3.91958500	-1.61000900	-1.36726400
H	-2.21519000	-1.08386300	-1.46637800
H	-2.75338200	-2.10305200	-0.10684800
C	-3.80851600	1.11100000	-0.93473900
H	-2.98595600	1.34540200	-1.62458500
H	-4.68570700	0.80447300	-1.52453700
H	-4.06235900	2.02219500	-0.37550400

$C(CN)_3^-$:

C	-2.17062600	0.27922900	0.77522900
C	-3.06593400	-0.31553200	-0.14665000
C	-3.24539000	0.25697900	-1.42929200
C	-3.78205900	-1.48260600	0.21426100
N	-4.37048500	-2.44152200	0.51078000
N	-3.39287300	0.72735900	-2.48318300
N	-1.43497000	0.76801800	1.53258300

5. References

[1] D. Ergöçmen, J. M. Goicoechea, *Angew. Chem. Int. Ed.* **2021**, *60*, 25286−25289.

[2] F. Thétiot, S. Triki, J. S. Pala, C. J. Gómez-García, *J. Chem. Soc., Dalton Trans.* **2002**, 1687−1693.

[3] L. Tendera, M. Helm, M. J. Krahfuss, M. W. Kuntze-Fechner, U. Radius, *Chem. Eur. J.* **2021**, *27*, 17849−17861.

[4] M. Brookhart, B. Grant, A. F. Volpe, *Organometallics* **1992**, *11*, 3920−3922.

[5] *CrysAlisPro*, Agilent Technologies, Version 1.171.35.8.

[6] (a) G. M. Sheldrick in SHELXL97, Programs for Crystal Structure Analysis (Release 97-2), Institut für Anorganische Chemie der Universität, Tammanstrasse 4, D-3400 Göttingen, Germany, 1998; (b) G. M. Sheldrick, *Acta Crystallogr. Sect. A* **1990**, *46*, 467−473; (c) G. M. Sheldrick, *Acta Crystallogr. Sect. A* **2008**, *64*, 112−122.

[7] M. J. Frisch, G. W. Trucks, H. B. Schlegel, G. E. Scuseria, M. A. Robb, J. R. Cheeseman, G. Scalmani, V. Barone, G. A. Petersson, H. Nakatsuji, X. Li, M. Caricato, A. V. Marenich, J. Bloino, B. G. Janesko, R. Gomperts, B. Mennucci, H. P. Hratchian, J. V. Ortiz, A. F. Izmaylov, J. L. Sonnenberg, Williams, F. Ding, F. Lipparini, F. Egidi, J. Goings, B. Peng, A. Petrone, T. Henderson, D. Ranasinghe, V. G. Zakrzewski, J. Gao, N. Rega, G. Zheng, W. Liang, M. Hada, M. Ehara, K. Toyota, R. Fukuda, J. Hasegawa, M. Ishida, T. Nakajima, Y. Honda, O. Kitao, H. Nakai, T. Vreven, K. Throssell, J. A. Montgomery Jr., J. E. Peralta, F. Ogliaro, M. J. Bearpark, J. J. Heyd, E. N. Brothers, K. N. Kudin, V. N. Staroverov, T. A. Keith, R. Kobayashi, J. Normand, K. Raghavachari, A. P. Rendell, J. C. Burant, S. S. Iyengar, J. Tomasi, M. Cossi, J. M. Millam, M. Klene, C. Adamo, R. Cammi, J. W. Ochterski, R. L. Martin, K. Morokuma, O. Farkas, J. B. Foresman, D. J. Fox, Wallingford, CT, **2016**.

[8] Y. Zhao, D. G. Truhlar, *Theor. Chem. Acc.* **2008**, *120*, 215−241.

[9] F. Weigend, *Phys. Chem. Chem. Phys.* **2006**, *8*, 1057−1065.

[10] F. Weigend, R. Ahlrichs, *Phys. Chem. Chem. Phys.* **2005**, *7*, 3297−3305.

[11] A. Bondi, *J. Phys. Chem.* **1964**, *68*.

[12] E. D. Glendening, J. K. Badenhoop, A. E. Reed, J. E. Carpenter, J. A. Bohmann, C. M. Morales, P. Karafiloglou, C. R. Landis, F. Weinhold, NBO 7.0, University of Wisconsin: Madison, WI, 2018.

[13] J. P. Perdew, K. Burke, M. Ernzerhof, *Phys. Rev. Lett.* **1997**, *78*, 1396−1396.

[14] J. P. Perdew, K. Burke, M. Ernzerhof, *Phys. Rev. Lett.* **1996**, *77*, 3865−3868.

[15] C. Adamo, V. Barone, *J. Chem. Phys.* **1999**, *110*, 6158−6170.

[16] A. D. McLean, G. S. Chandler, *J. Chem. Phys.* **1980**, *72*, 5639−5648.

[17] R. Krishnan, J. S. Binkley, R. Seeger, J. A. Pople, *J. Chem. Phys.* **1980**, *72*, 650−654.

[18] G. Becker, G. Gresser, W. Uhl, *Z. Naturforsch. B* **1981**, *36*, 16−19.

[19] Chemcraft − Graphical Software for Visualization of Quantum Chemistry Computations. https://www.chemcraftprog.com
